# Supplementary material for: Cysteinyl-tRNA synthetase governs cysteine polysulfidation and mitochondrial bioenergetics
Source: Nat Commun. 2017 Oct 27;8:1177. doi: 10.1038/s41467-017-01311-y (PMC5660078; doi:10.1038/s41467-017-01311-y)
Supplement: Supplementary file 1 — Supplementary Information [file 41467_2017_1311_MOESM1_ESM.pdf]

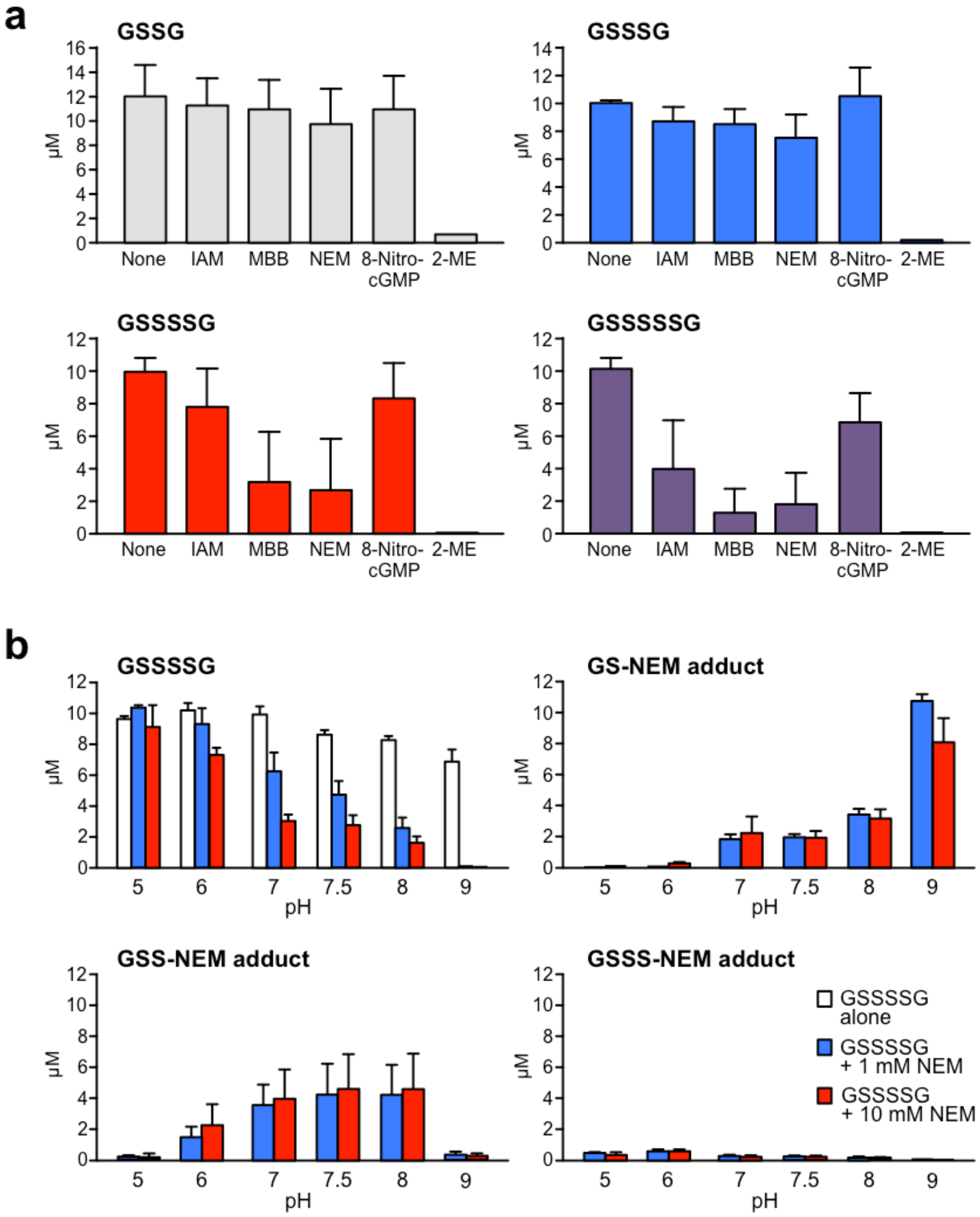

Supplementary Fig. 1. Nucleophilic and electrophilic reactions of glutathione polysulfides. (**a**) Decomposition of glutathione polysulfides with various electrophiles and a nucleophile (reducing agent), which indicates that polysulfides possess mixed sulfurs—both nucleophilic and electrophilic characteristics. Oxidized glutathione (GSSG) and oxidized forms of glutathione polysulfides (GS-$(S)_n$-SG, n = 1−3) (10 μM) were incubated with various electrophiles or 2-mercaptoethanol (2-ME; 5 mM each) in 20 mM Tris-HCl pH 7.5 at 37 °C for 1 h, and they were then analyzed with LC-ESI-MS/MS. Electrophiles used included iodoacetamide (IAM), monobromobimane (MBB), *N*-ethylmaleimide (NEM), and 8-nitroguanosine 3',5'-cyclic monophosphate (8-nitro-cGMP). (**b**) Decomposition of oxidized glutathione

tetrasulfide (GSSSSG) with NEM and production of NEM adducts. GSSSSG (10 μM) was treated with NEM (1 or 10 mM) in 20 mM Tris-HCl or phosphate buffer (pH 5−9) at 37 °C for 1 h, followed by LC-ESI-MS/MS measurement of decomposed GSSSSG and NEM adducts. Data are means ± s.d. (*n* = 3). Whereas a simple disulfide (GSSG) is electrophilic, certain sulfur residues of GS-$(S)_n$-SG were nucleophilic; nevertheless, all GS-$(S)_n$-SG compounds tested decomposed in the reaction with various electrophiles.

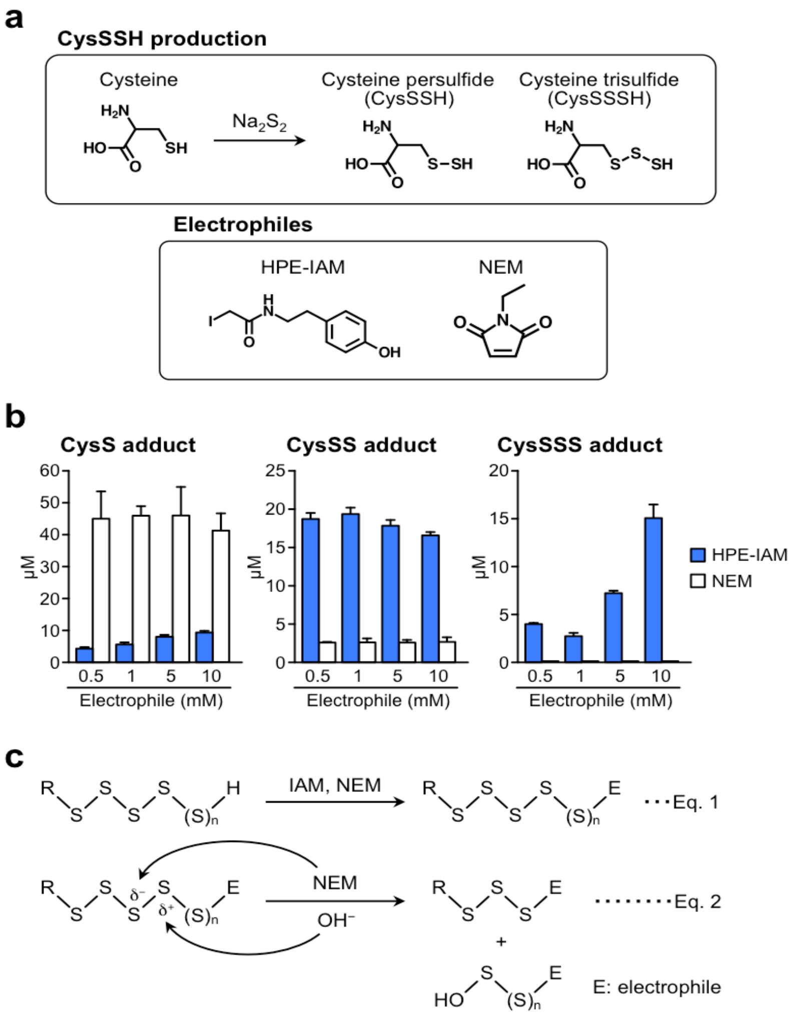

Supplementary Fig. 2. Chemical reactivity of cysteine hydropolysulfides (CysS-$(S)_n$-H). (**a**) Chemical reaction producing CysS-$(S)_n$-H and structures of electrophiles, such as HPE-IAM and NEM. (**b**) Different profiles of production of electrophile-sulfide/polysulfide adducts in the reaction of CysS-$(S)_n$-H with electrophiles (HPE-IAM and NEM). In the reaction illustrated in the upper panel in **a**, cysteine persulfide (CysSSH) and cysteine trisulfide (CysSSSH) formed from cysteine (50 μM) and Na_2S_2 (150 μM) in 30 mM HEPES buffer pH 7.5 at 37 °C for 5 min, followed by reaction with 0.5, 1, 5, or 10 mM HPE-IAM or NEM at 37 °C for 1 h, after which adducts were measured via LC-ESI-MS/MS. Data are means ± s.d. (n = 3). (**c**) Schematic representation of the polysulfide reaction with electrophiles. Whereas the weak electrophile IAM mainly reacts with the hydrosulfide moiety of CysS-$(S)_n$-H to form stable polysulfide adducts, strong electrophiles such as NEM undergo repeated alkylating reactions to finally produce simple monothiol adducts during reactions with CysS-$(S)_n$-H.

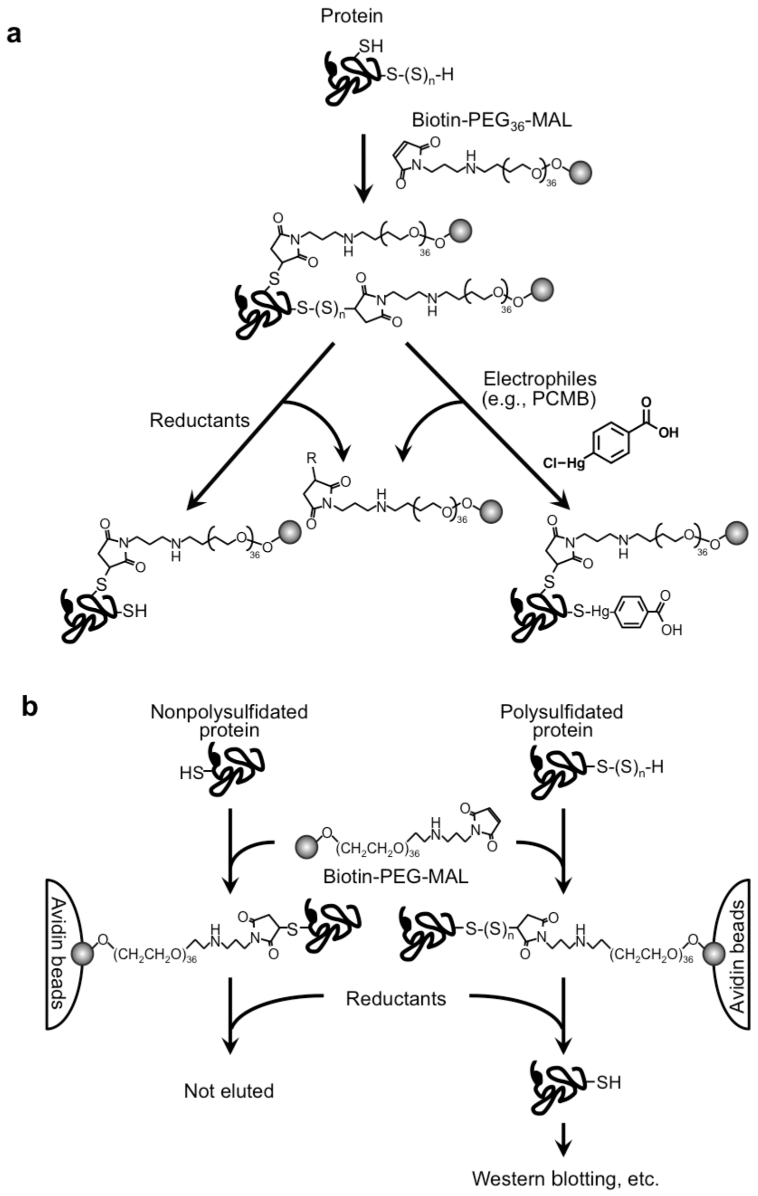

Supplementary Fig. 3. Detection of CysS-(S)$_n$-H in various proteins. Schematic illustration of the biotin-polyethylene glycol (PEG)-conjugated maleimide (biotin-PEG-MAL) labeling gel shift assay (PMSA) for identification of polysulfidated proteins by using biotin-PEG 36-mer (PEG$_{36}$)-conjugated maleimide (biotin-PEG$_{36}$-MAL) and different electrophilic compounds, e.g., *p*-chloromercuribenzoic acid (PCMB) (**a**), and application to the biotin-PEG$_{36}$-MAL capture method, represented in **b**, for quantitative identification of endogenous polysulfidated proteins, which were isolated by reductive treatment of biotin-PEG$_{36}$-MAL-bound avidin beads that captured polysulfidated proteins, followed by specific detection with Western blotting (**b**).

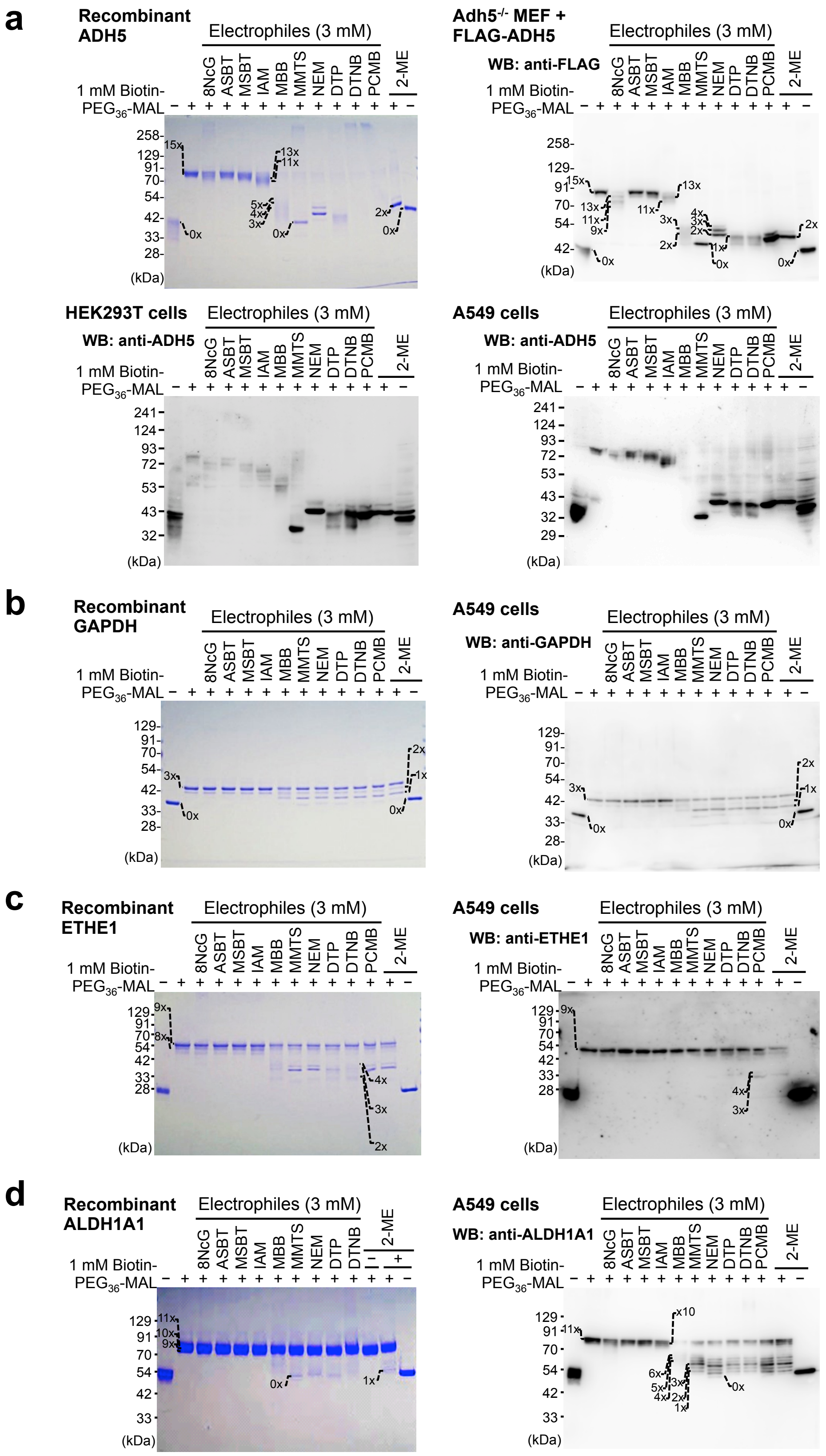

Supplementary Fig. 4. Identification of polysulfidated proteins by PMSA. Extensive polysulfidation was observed with various recombinant and endogenous

proteins in cultured cells. Protein bands of ADH5 (**a**), GAPDH (**b**), ethylmalonic encephalopathy protein 1 (ETHE1, **c**), and aldehyde dehydrogenase A family, member A1 (ALDH1A1, **d**) were detected by using Coomassie blue stain (SDS-PAGE) for recombinant proteins, prepared in an *E. coli* expression system (Supplementary Table 1), and by Western blotting (WB) with respective cell lysates. Electrophiles used included 8-nitro-cGMP (8NcG), 2-aminosulfonyl benzothiazole (ASBT), 2-methylsulfonyl benzothiazole (MSBT), IAM, MBB, methyl methanethiosulfonate (MMTS), NEM, 4,4'-dithiopyridine (DTP), 5'-dithiobis(2-nitrobenzoic acid) (DTNB), and PCMB. Multiple numbers shown in each panel indicate degrees of biotin-PEG_{36}-MAL labeling in protein bands.

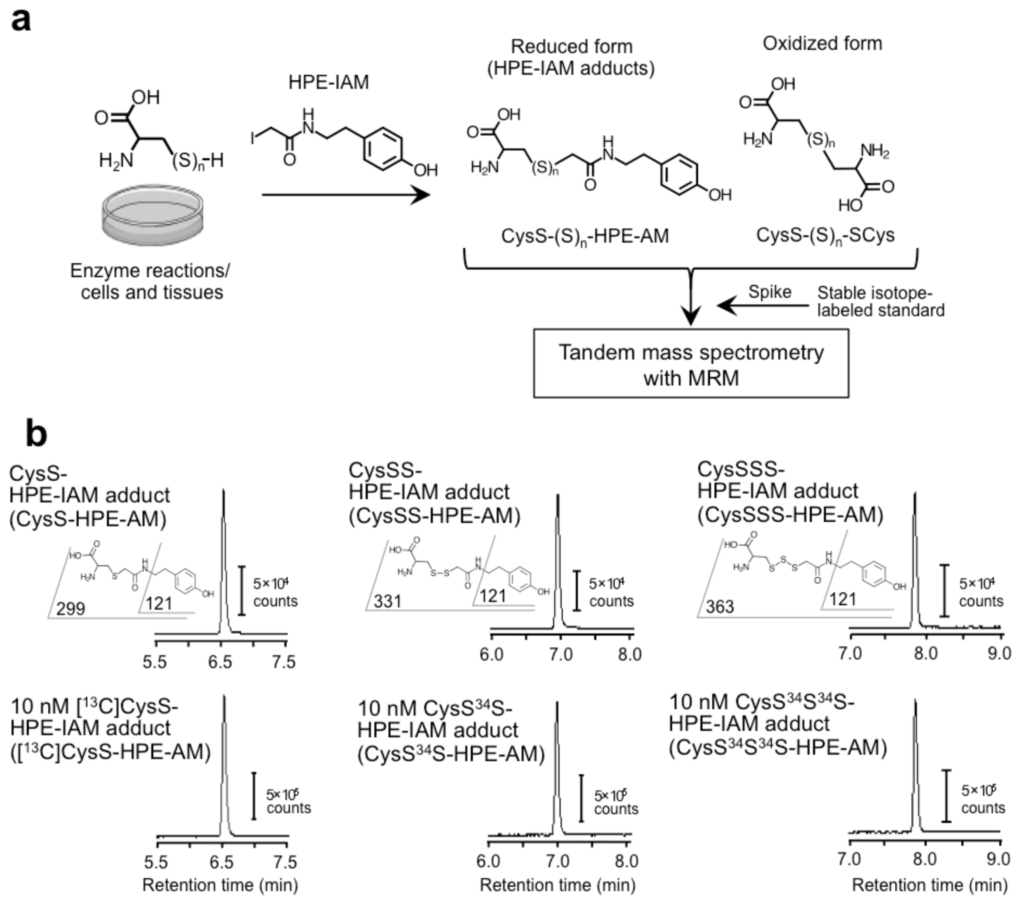

Supplementary Fig. 5. Schematic representation of the LC-ESI-MS/MS approach with HPE-IAM trapping (a) and LC-ESI-MS/MS profiles of the CysS-$(S)_n$-H standard for each HPE-IAM adduct (b). Cysteine (CysSH), CysS-$(S)_n$-H (i.e., CysSSH and CysSSSH), and other related sulfide compounds were derivatized with HPE-IAM, whose MRM parameters appear in Supplementary Table 2, and were used for our present LC-ESI-MS/MS analysis.

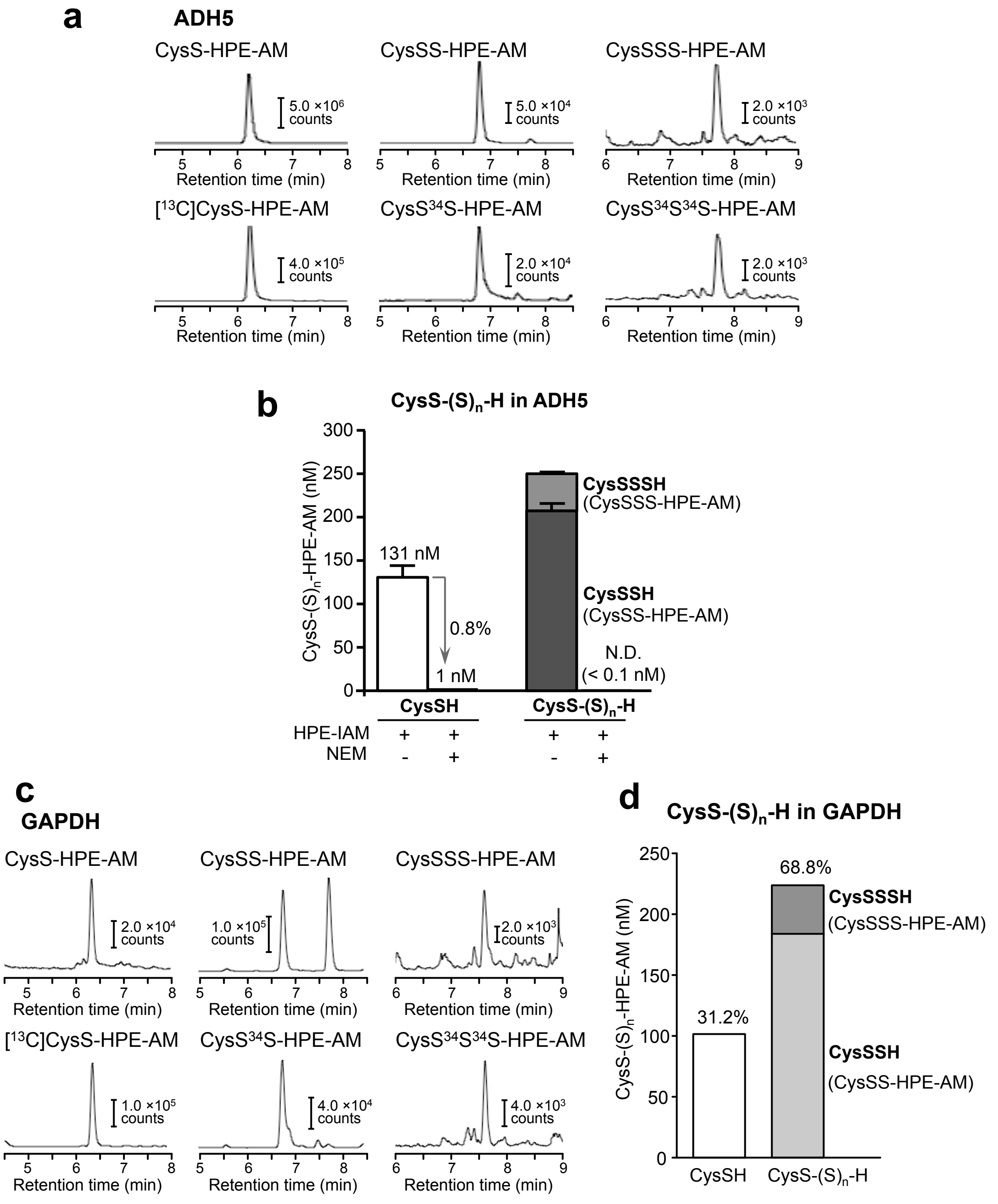

Supplementary Fig. 6. CysS-$(S)_n$-H formation (polysulfidation) in various proteins. (**a**) LC-ESI-MS/MS chromatograms obtained from analysis of CysS-$(S)_n$-H formation in recombinant ADH5, quantitatively represented in Fig. 1a. (**b**) Effects of NEM treatment of the amounts of CysS-$(S)_n$-H detected in ADH5. Recombinant ADH5 (0.85 mg/ml) was alkylated with 6 mM HPE-IAM and 60 mM NEM at 37 °C for 5 min and was digested by 1 mg/ml Pronase, in 40 mM sodium acetate buffer (pH 5.5) in the presence of known amounts of isotope-labeled internal standards at 37 °C for 7 h, to produce cysteine or CysS-$(S)_n$-H. After addition of 0.1% formic acid and

centrifugation, the supernatants were subjected to LC-ESI-MS/MS. (**c**) CysS-$(S)_n$-H LC-ESI-MS/MS chromatograms for GAPDH, and (**d**) their quantitative representation. The amounts of CysS-$(S)_n$-H formed in both proteins were quantified based on the signal intensity (lower panels in **a** and **c**) of the known amounts of stable isotope-labeled internal standards spiked before pronase digestion.

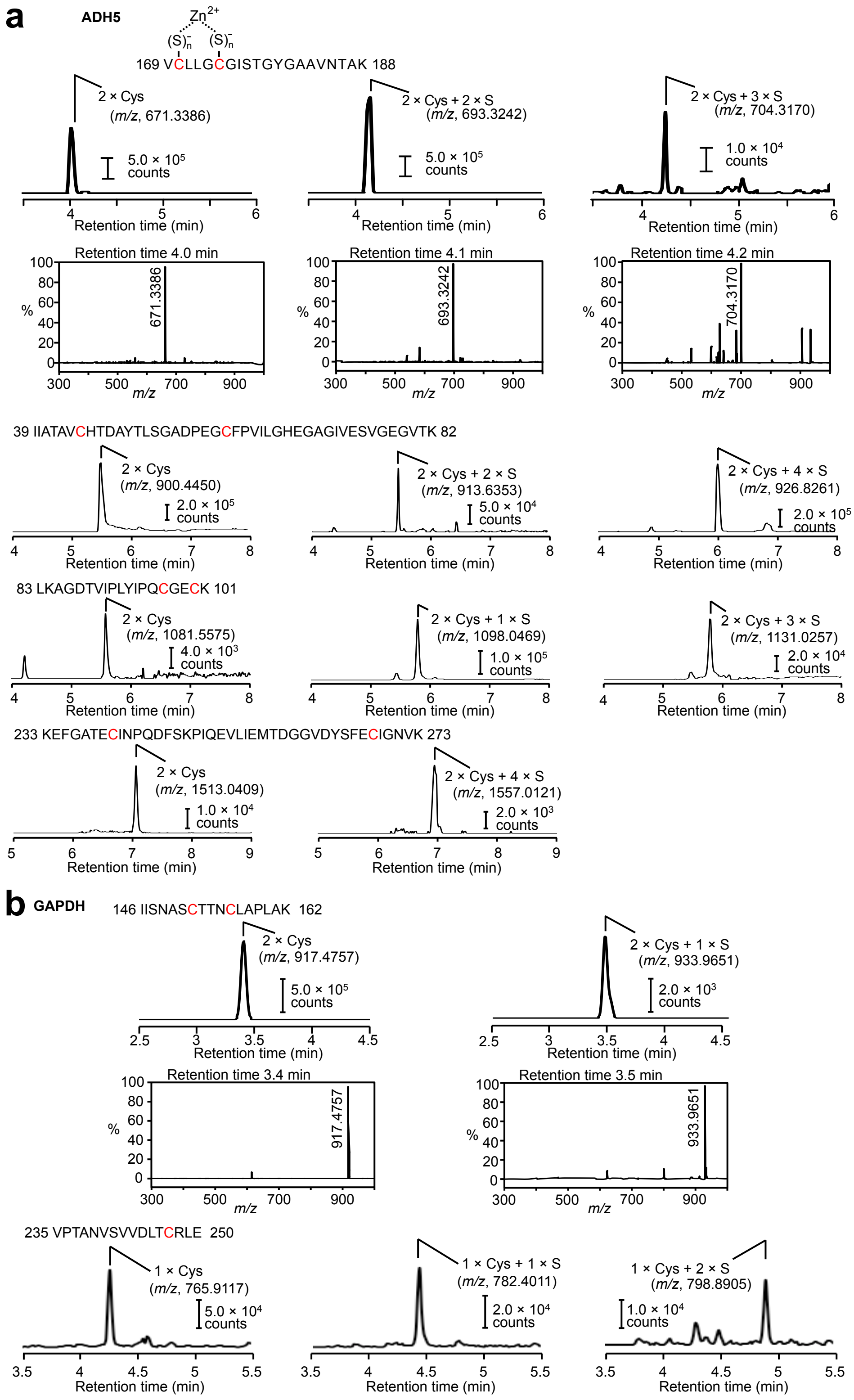

Supplementary Fig. 7. LC-Q-TOF-MS detection of polysulfidation sites in recombinant ADH5 (a) and GAPDH (b). Extracted-ion chromatograms and MS

spectra are presented for peptide fragments containing carbamidomethyl-cysteine/cysteine polysulfide residues with their *m/z* values for ADH5 (**a**) and GAPDH (**b**). Polysulfidation occurred on 8 cysteine residues (of 15 cysteine residues of ADH5) (**a**) and 2 of 3 cysteine residues of GAPDH (**b**), which were analyzed by MS/Mascot.

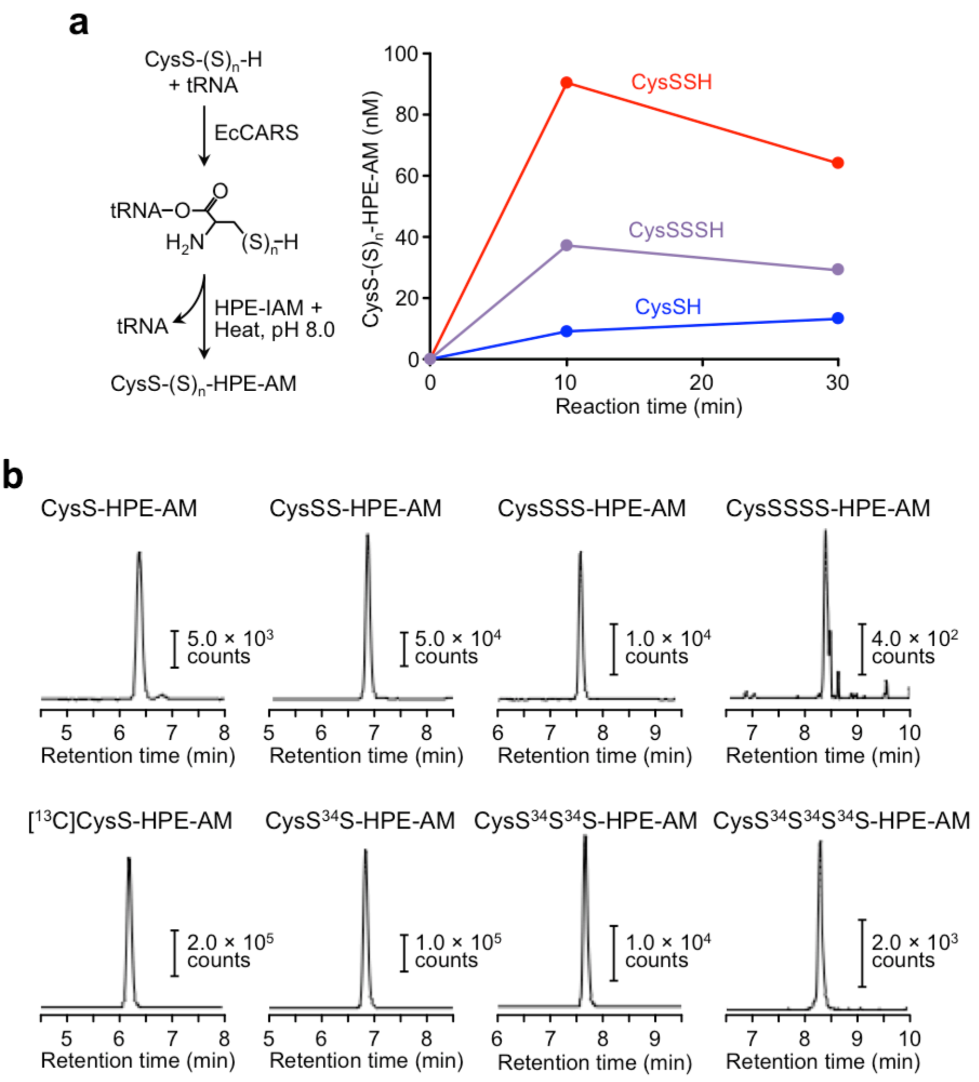

Supplementary Fig. 8. CysS-$(S)_n$-H production and incorporation into Cys-tRNA by EcCARS. (**a**) Schematic illustration showing Cys-tRNA biosynthesis from CysS-$(S)_n$-H as substrates, with catalysis by EcCARS (left panel). CysSSH and CysSSSH formed from cystine and Na_2S_2 were reacted with EcCARS in the presence of Cys-tRNA, followed by identification of polysulfide-bound Cys-tRNA by LC-ESI-MS/MS analysis (right panel). (**b**) LC-ESI-MS/MS chromatograms obtained from analyses of CysS-$(S)_n$-H production and incorporation into Cys-tRNA catalyzed by EcCARS with cysteine as the substrate; Fig. 1b provides quantitative data.

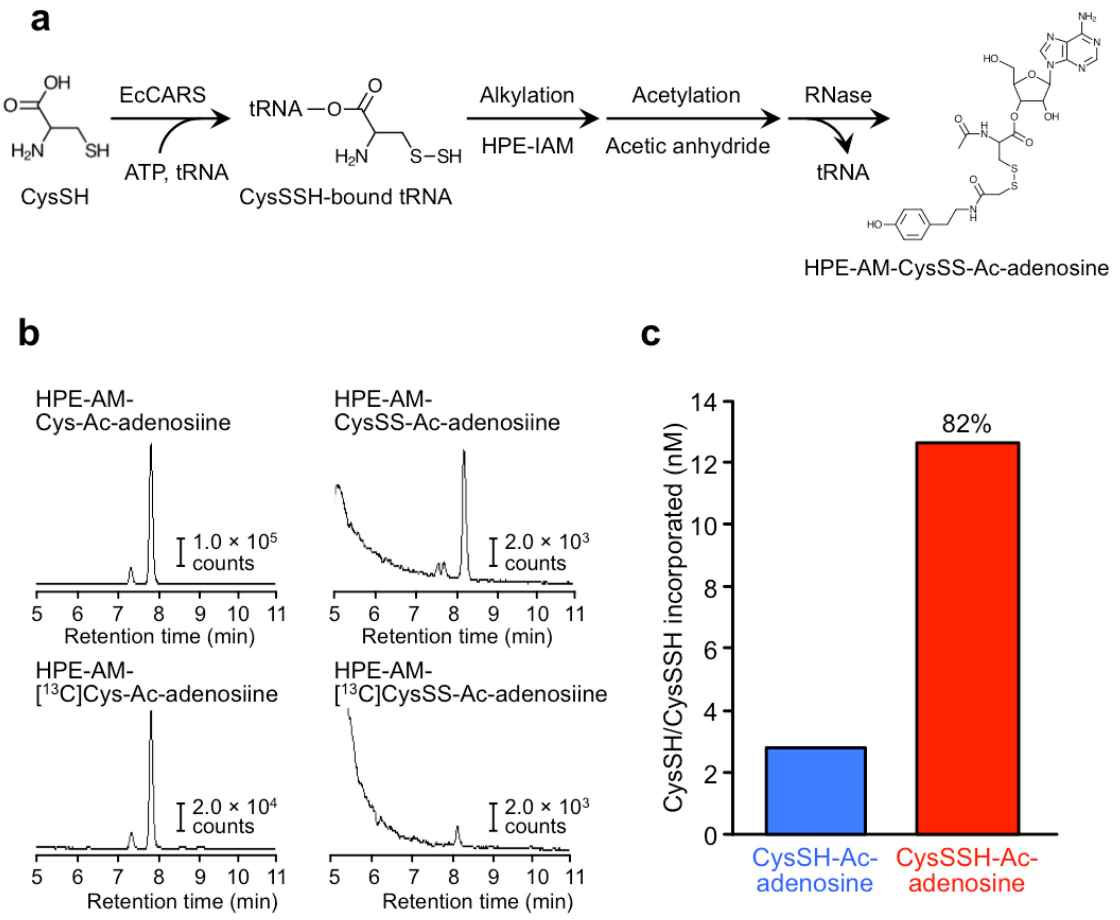

Supplementary Fig. 9. LC-ESI-MS/MS analysis for CysSSH-bound Cys-tRNA (Cys-tRNA^{CysSSH}). (**a**) Schematic diagram of the method of *de novo* synthesis of CysSSH-bound Cys-tRNA (Cys-tRNA^{CysSSH}), as catalyzed by EcCARS, and its nuclease (RNase) digest to obtain HPE-AM-CysSS-Ac-adenosine, which is an HPE-IAM-persulfide adduct of acetylated cysteine (HPE-AM-CysSS-Ac) bound to adenosine. (**b**) Typical LC-ESI-MS/MS chromatograms obtained. (**c**) LC-ESI-MS/MS quantification of a CysS-(S)$_n$-H-adenosine adduct (CysSS-Ac-adenosine). CysSSH bound to Cys-tRNA, synthesized via the reaction of EcCARS with cysteine, was directly quantified as an HPE-IAM adduct of acetylated cysteine persulfide bound to adenosine (HPE-AM-CysSS-Ac-adenosine) generated from the RNase digest of the Cys-tRNA^{CysSSH} after alkylation (HPE-IAM) and acetylation (acetic anhydride) of the CysSSH residue, shown in panel **a**.

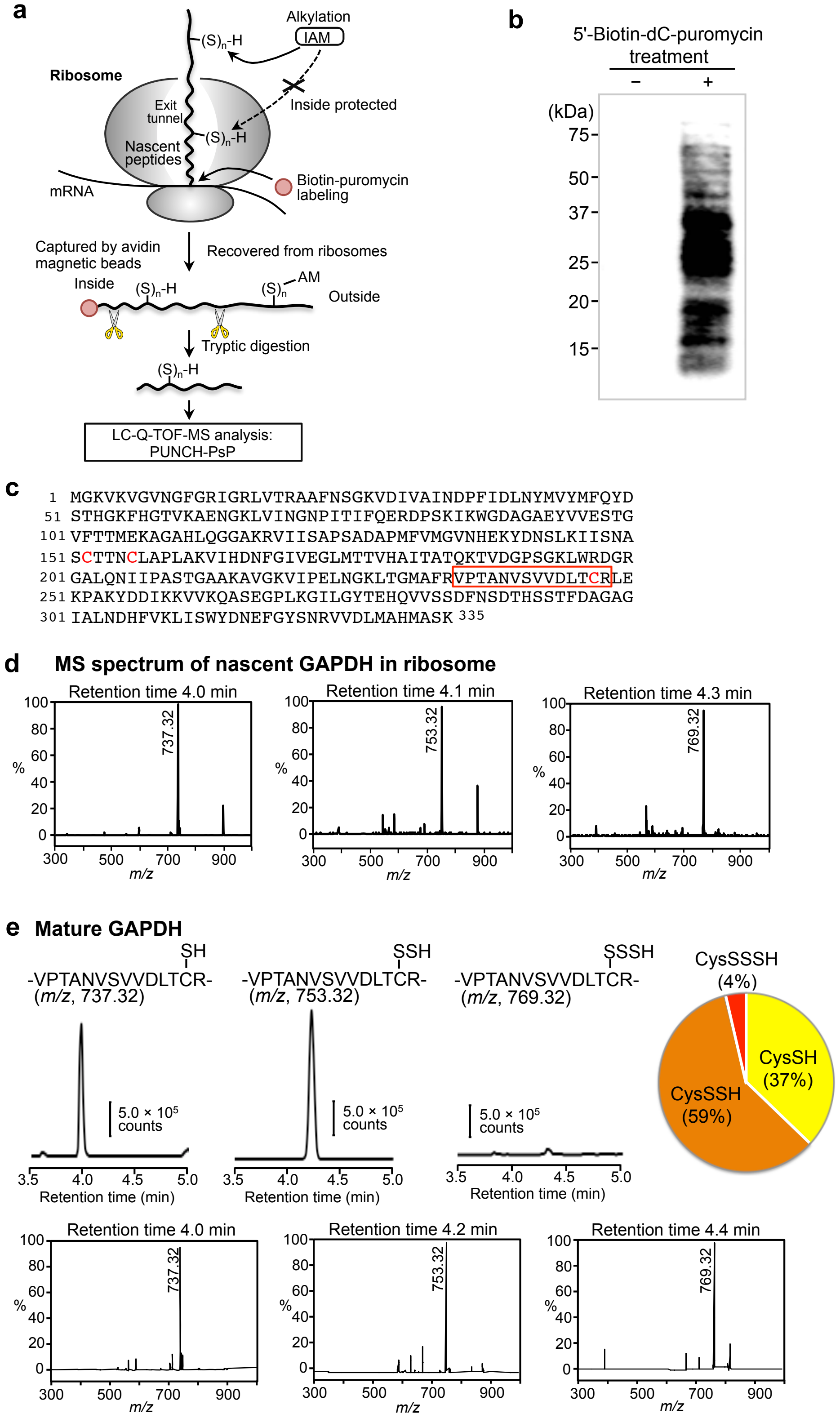

Supplementary Fig. 10. Identification of CysS-(S)$_n$-H-bound nascent GAPDH polypeptides formed endogenously in ribosomes and in the mature GAPDH

protein. (**a**) Schematic illustration of a new method for detection of polysulfide-bound nascent polypeptides, i.e., PUNCH-PsP, which we developed and successfully applied to specific identification of CysS-$(S)_n$-H-containing polypeptides formed endogenously and present in the polypeptide exit tunnel of the ribosome. Because CysS-$(S)_n$-H residues that are extruded and exposed outside the ribosome are readily alkylated with IAM or chemically modified, the CysS-$(S)_n$-H residues of polypeptides that are newly synthesized and thus remain inside the polypeptide exit tunnel of the ribosome can be identified as the non-alkylated forms of the hydropolysulfides, as Fig. 1c shows. (**b**) Western blotting (streptavidin-peroxidase labeling) of nascent polypeptides *de novo* synthesized in ribosomes recovered from *E. coli* cells after transfection of the pGE30-hGAPDH-expressing vector used for this biotin-puromycin-avidin capture method as illustrated in **a**. Puromycin-labeled (biotinylated) nascent polypeptides were detected with ribosomes isolated from *E. coli* cells by Western blotting via the streptavidin-conjugated peroxidase reaction: 0.89 μg ribosomal protein with or without 5'-biotin-dC-puromycin treatment was applied to each lane. (**c**) The amino acid sequence of hGAPDH, with three cysteine residues being marked in red and the CysS-$(S)_n$-H-containing peptide sequence identified by PUNCH-PsP, indicated by a red box. (**d**) MS spectra obtained from analysis of CysS-$(S)_n$-H formation in nascent GAPDH, represented in Fig. 1c. (**e**) Direct identification by LC-Q-TOF of native forms of CysSH, CysSSH, and CySSSH residues present in mature GAPDH protein. Extracted-ion chromatograms and MS spectra are presented for peptide fragments containing cysteine/cysteine polysulfide residues with their *m/z* values for mature GAPDH. As soon as the recombinant GAPDH was isolated from *E. coli*, followed by quick digestion with trypsin, which was promptly subjected to the LC-ESI-Q-TOF analysis, in a similar manner as shown for the PUNCH-PsP method.

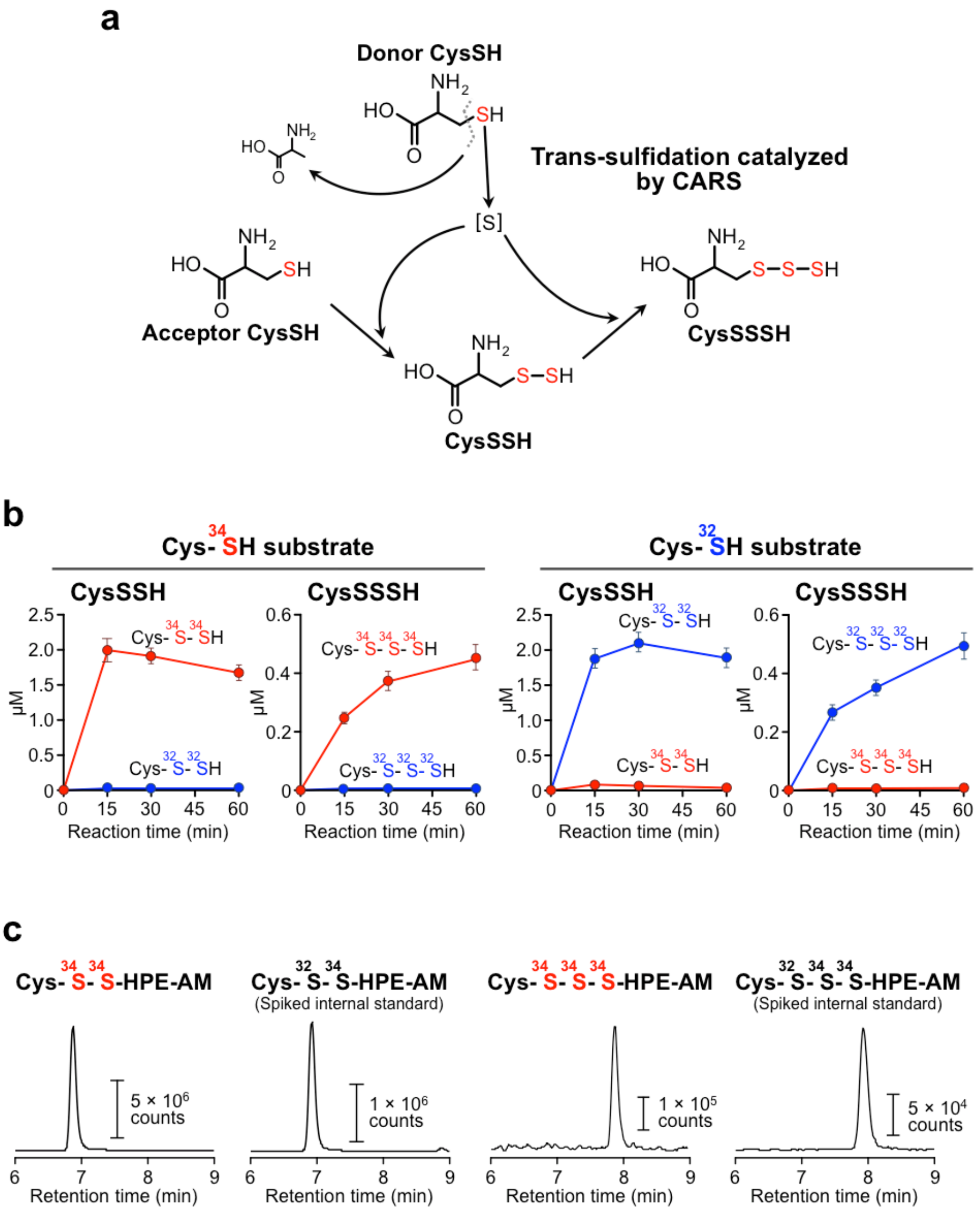

Supplementary Fig. 11. Stable isotope (^{34}S) tracer analysis of CysSSH formation from cysteine (CysSH) (sulfur transfer reaction), as catalyzed by EcCARS. To clarify the molecular mechanism of CysSSH and CysSSSH formation from CysSH, 50 μM ^{34}S-labeled L-cysteine (Cys-^{34}SH) was reacted with 200 μg/ml EcCARS at 37 °C for 15-60 min, after which the mixtures were treated with 1 mM HPE-IAM, followed by LC-ESI-MS/MS. (**a**) Schematic diagram of the EcCARS-catalyzed reaction to produce CysSSH and CysSSSH from cysteine (CysSH), in which a sulfur is cleaved from the donor cysteine and transferred to the acceptor cysteine thiol. (**b**) Quantitative representation of the results of an EcCARS reaction with 50 μM Cys-^{34}SH (left two panels) or Cys-^{32}SH (right two panels). Data are means ± s.d. (n = 3). (**c**)

LC-ESI-MS/MS chromatograms for the reactions shown in the left panels in **b** for Cys-^{34}S-^{34}S-HPE-AM (MRM transition, 334.8 > 121.0; collision energy, -29 V), Cys-^{34}S-^{34}S-^{34}S-HPE-AM (MRM transition, 368.8 > 121.0; collision energy, -29 V), and each internal standard spiked in the EcCARS enzymatic reaction with Cys-^{34}SH as a substrate for 15 min.

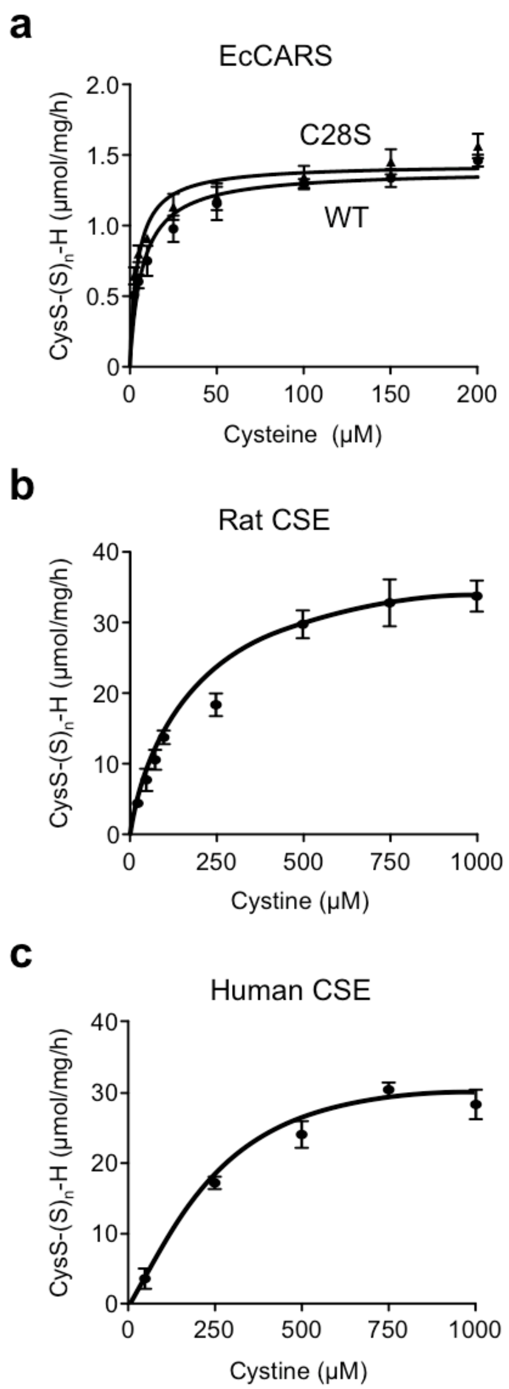

Supplementary Fig. 12. Enzyme kinetic analyses of CysS-(S)$_n$-H generation comparing wild-type (WT) EcCARS and its C28S mutant (a), recombinant rat CSE (b), and recombinant human CSE (c). Supplementary Table 3 provides the enzyme kinetic parameters. Data are means ± s.d. (n = 3).

a

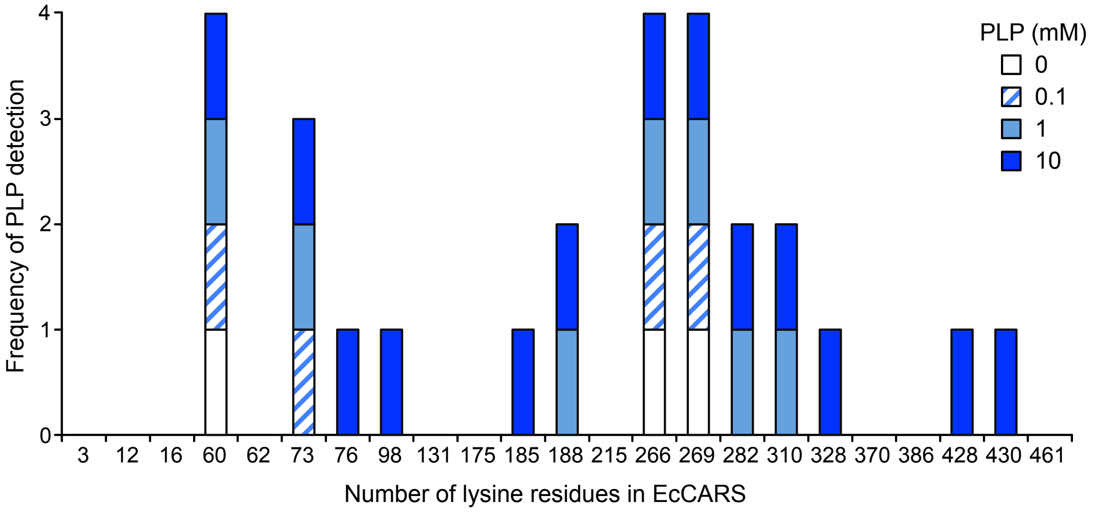

b

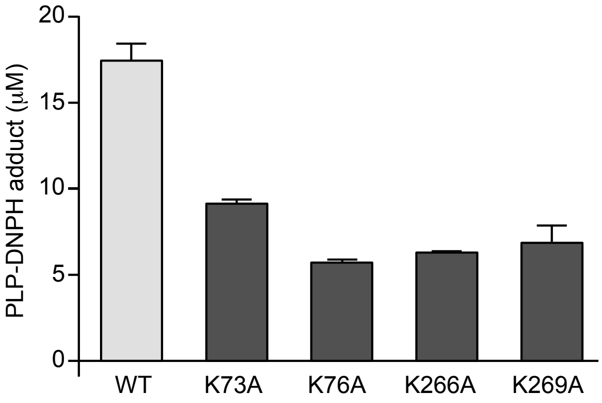

Supplementary Fig. 13. PLP binding of Lys residues in EcCARS identified by LC-Q-TOF-MS and Mascot data searches (a) and by DNPH-labeling LC-MS/MS analysis (b). (**a**) Recombinant EcCARS treated with various concentrations of PLP or not treated was digested with trypsin, and tryptic digests were subjected to LC-Q-TOF-MS combined with Mascot MS/MS data searches. The numbers (1−4) of detection frequency indicate the frequency of PLP detection of each Lys residue with increasing concentrations of PLP (0-10 mM). (**b**) The amount of PLP bound to EcCARS was quantified by LC-ESI-MS/MS analysis after PLP was extracted from various EcCARS proteins tested via the trapping reaction with DNPH. Wild-type and various Lys mutants of EcCARSs (15 μM each) were treated with 50 μM PLP at 37 °C for 1 h, after which free forms of PLP were completed eliminated with the PD SpinTrap G-25 column, and the protein fraction recovered was further reacted with 2 mM DNPH to form PLP-DNPH adduct at 37 °C for 1 h, followed by quantification by LC-ESI-MS/MS analysis.

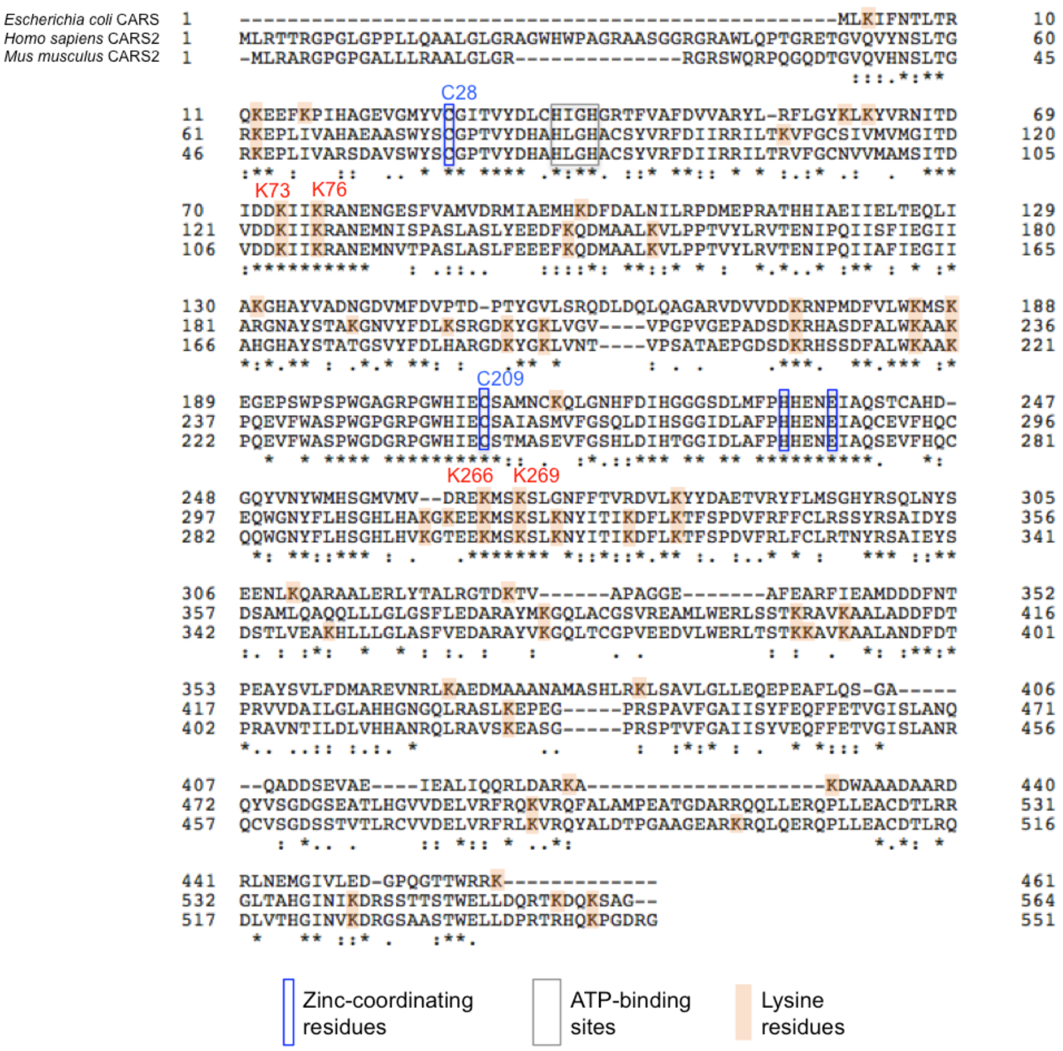

Supplementary Fig. 14. Amino acid sequence alignment of CARSs from different organisms including mammals.

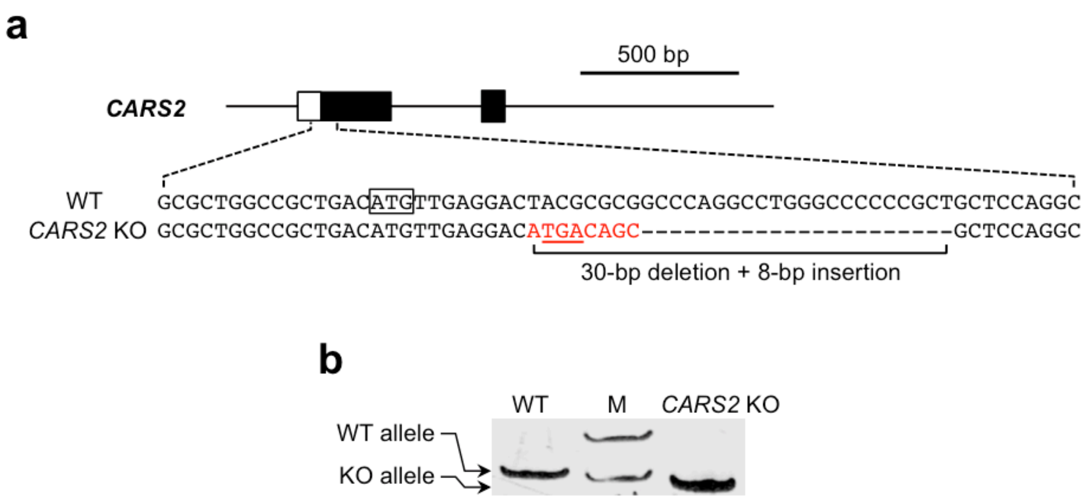

Supplementary Fig. 15. Generation of a *CARS2* KO cell line by using the CRISPR/Cas9 system. (**a**) Schematic illustration of the human *CARS2* gene structure and sequences of WT and mutant alleles around the target locus. A modified-allele sequence obtained from the *CARS2* targeted cell line is shown below. For the CRISPR/Cas9 system-mutagenized DNA sequences, deleted nucleotides are indicated by hyphens and inserted nucleotides are shown in red. The starting codon (ATG) is marked with a box. Because of the newly generated stop codon (TGA) in a mutant allele, as indicated in the modified allele sequence by an underline, the *CARS2* KO cell lines were expected to produce a nonfunctional premature N-peptide. (**b**) PCR analyses were performed with genomic DNAs from *CARS2* WT and homozygous *CARS2* KO HEK293T cells. A DNA molecular weight marker (M) shows 210-bp and 162-bp bands.

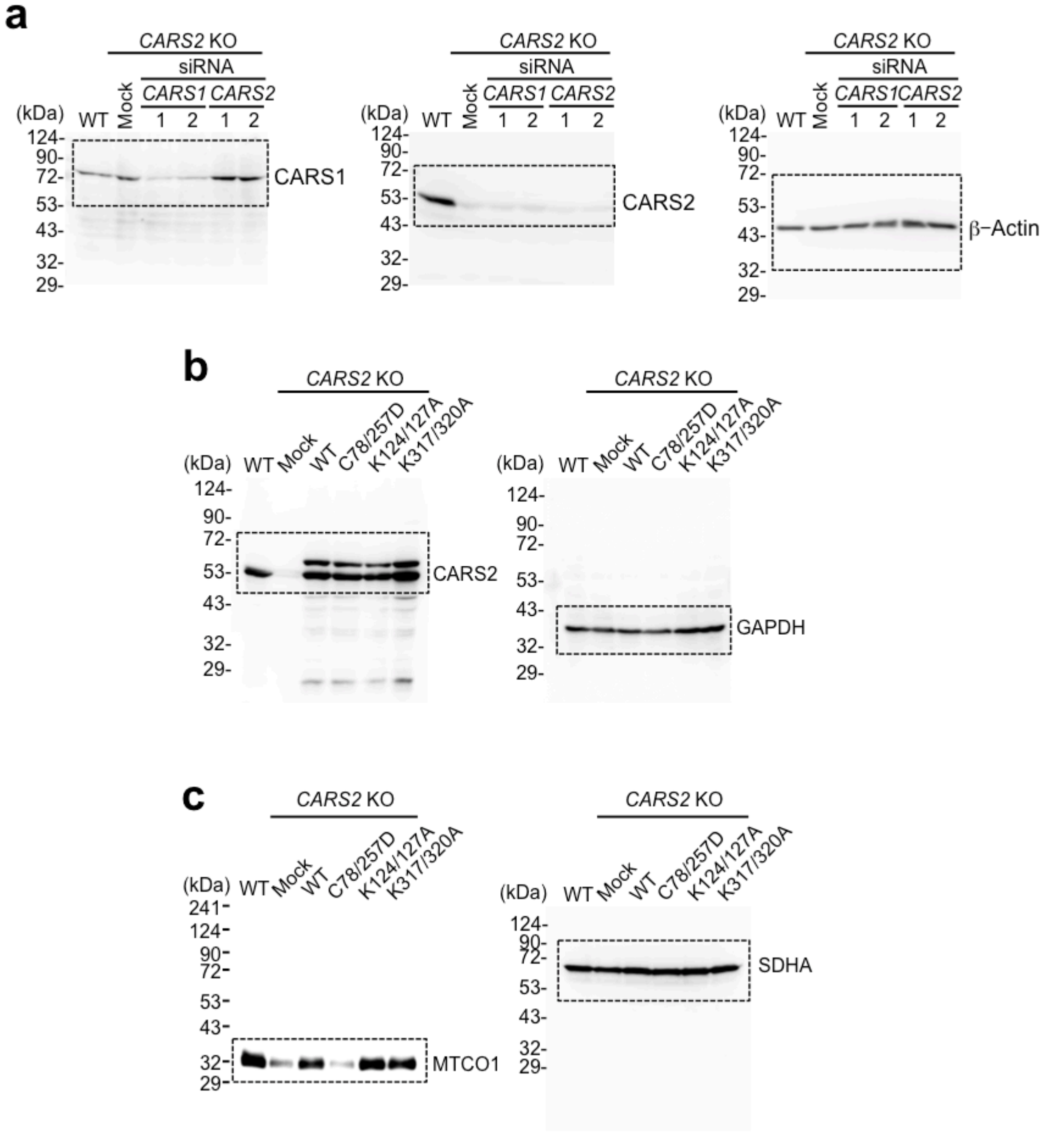

Supplementary Fig. 16. Uncropped images of the blots shown in Fig. 4.
(**a, b, c**) Full uncropped images of Figs. 4c, 4f, and 4g, respectively. Fig. 4 shows the areas marked by dashed lines.

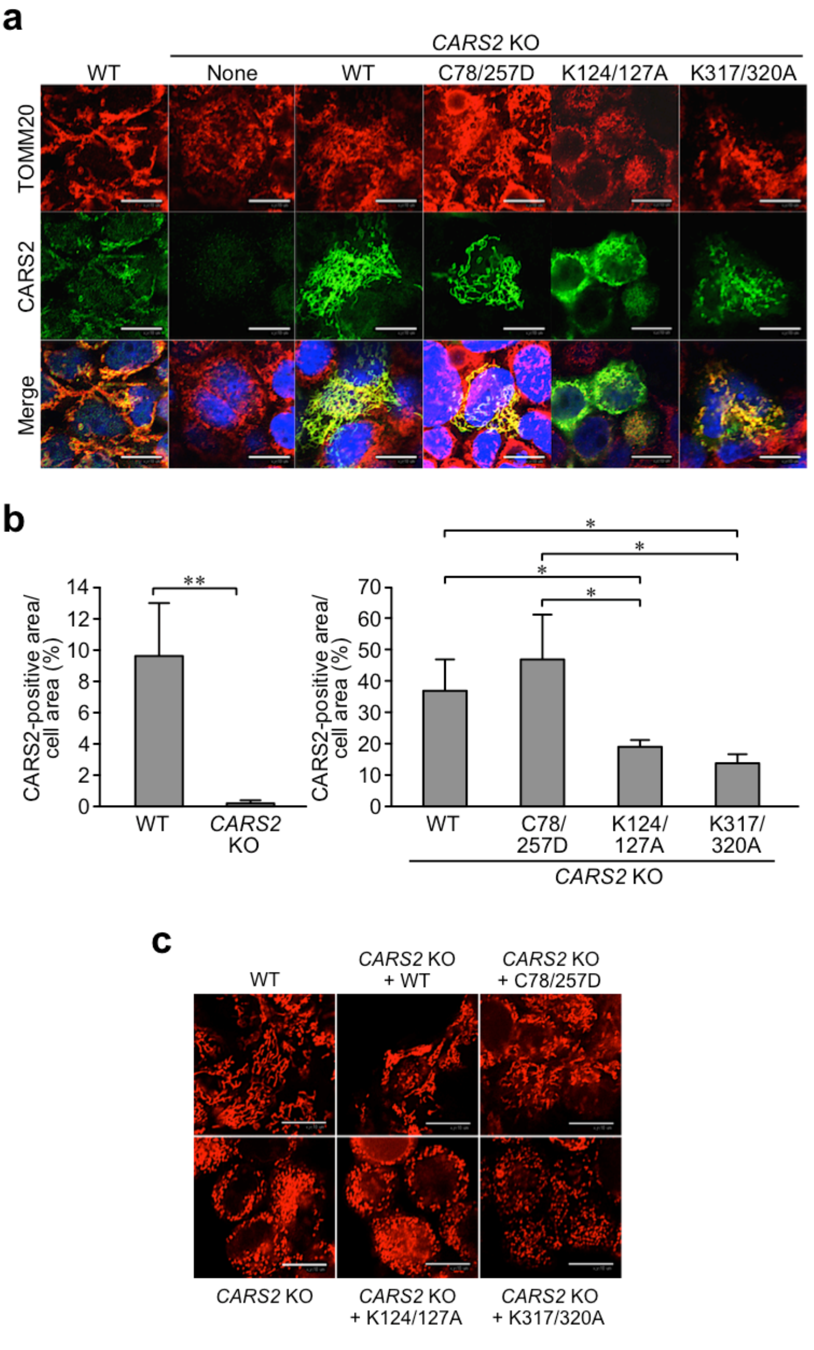

Supplementary Fig. 17. CARS2-dependent morphological dynamics of mitochondria in HEK293T cells. (**a**) Mitochondrial morphological analyses via immunofluorescence staining for TOMM20 and CARS2. (**b**) CARS2-positive cells detected in **a** were assessed morphometrically for WT and *CARS2* KO cells with or without *CARS2* WT or various mutants added back. Data are means ± s.d. ($n = 3$). *$P < 0.05$; **$P < 0.01$. (**c**) Mitochondrial morphological analyses via MitoTracker Red fluorescent mitochondrial stain. Scale bars, 10 µm. Fig. 8a gives the results of this MitoTracker Red imaging assessment.

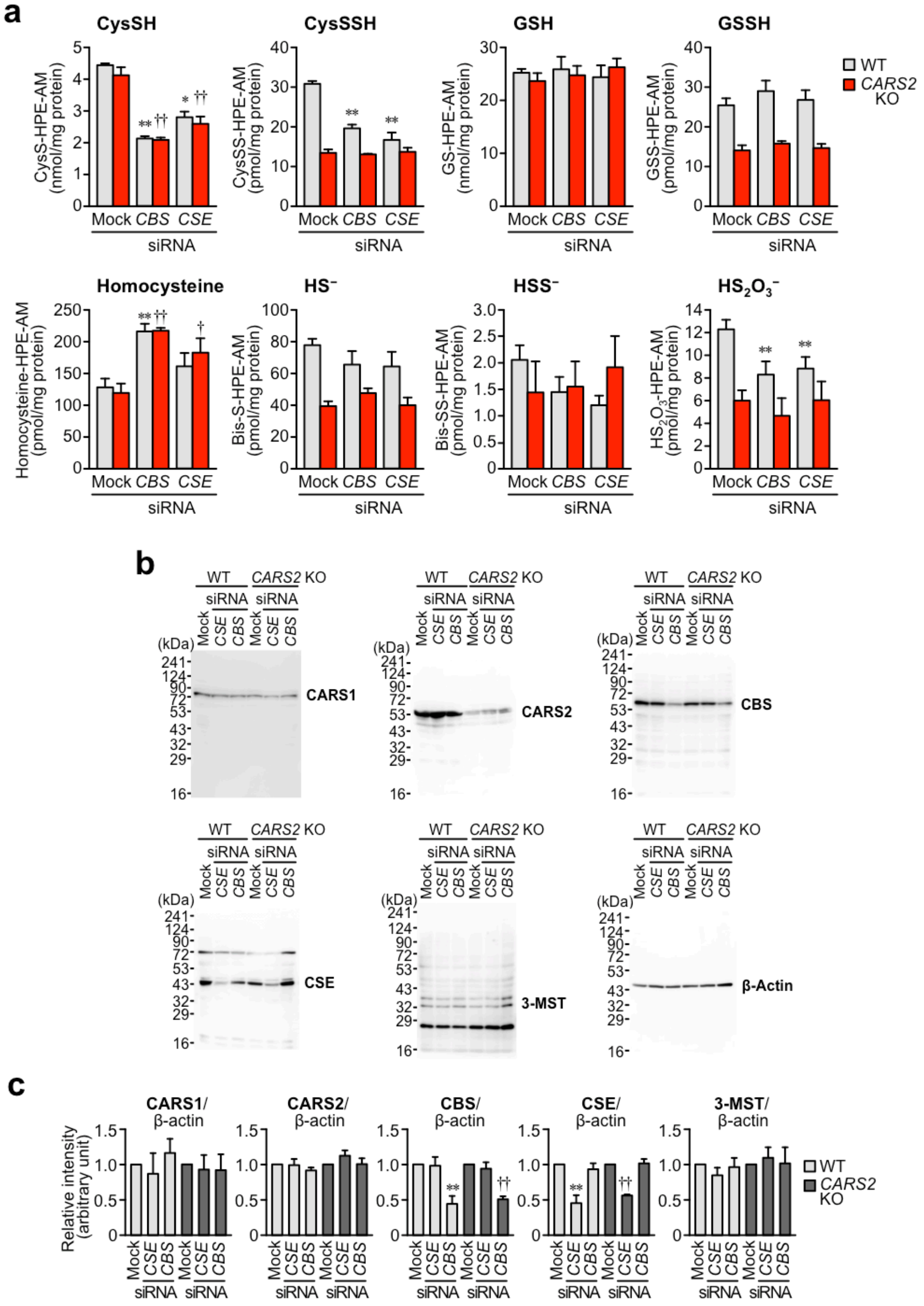

Supplementary Fig. 18. Endogenous formation of CysSSH and GSSH in HEK293T cells with or without CBS and CSE knockdown. Knockdown of CBS and CSE was performed as recently reported[1] by using the following small interfering RNAs (siRNAs): CBS, CBSHSS101428 (Invitrogen), and CSE, CTHHSS102447 (Invitrogen). siRNA transfection was performed by using Lipofectamine RNAiMAX

(Invitrogen) according to the manufacturer's instructions. (**a**) Intracellular levels of CysSSH, GSSH, and other related sulfide derivatives in WT and *CARS2* KO cells with CBS or CSE knocked down. (**b**) Western blotting of CARS1, CARS2, CBS, CSE, 3-MST, and β-actin in the cells used in **a**. (**c**) Relative immunoreactive band intensity determined by densitometric analysis of the Western blots (**b**) is shown. Data are means ± s.d. (n = 3). *P < 0.05 (vs. WT mock); **P < 0.01 (vs. WT mock); †P < 0.05 (vs. *CARS2* KO mock); ††P < 0.01 (vs. *CARS2* KO mock).

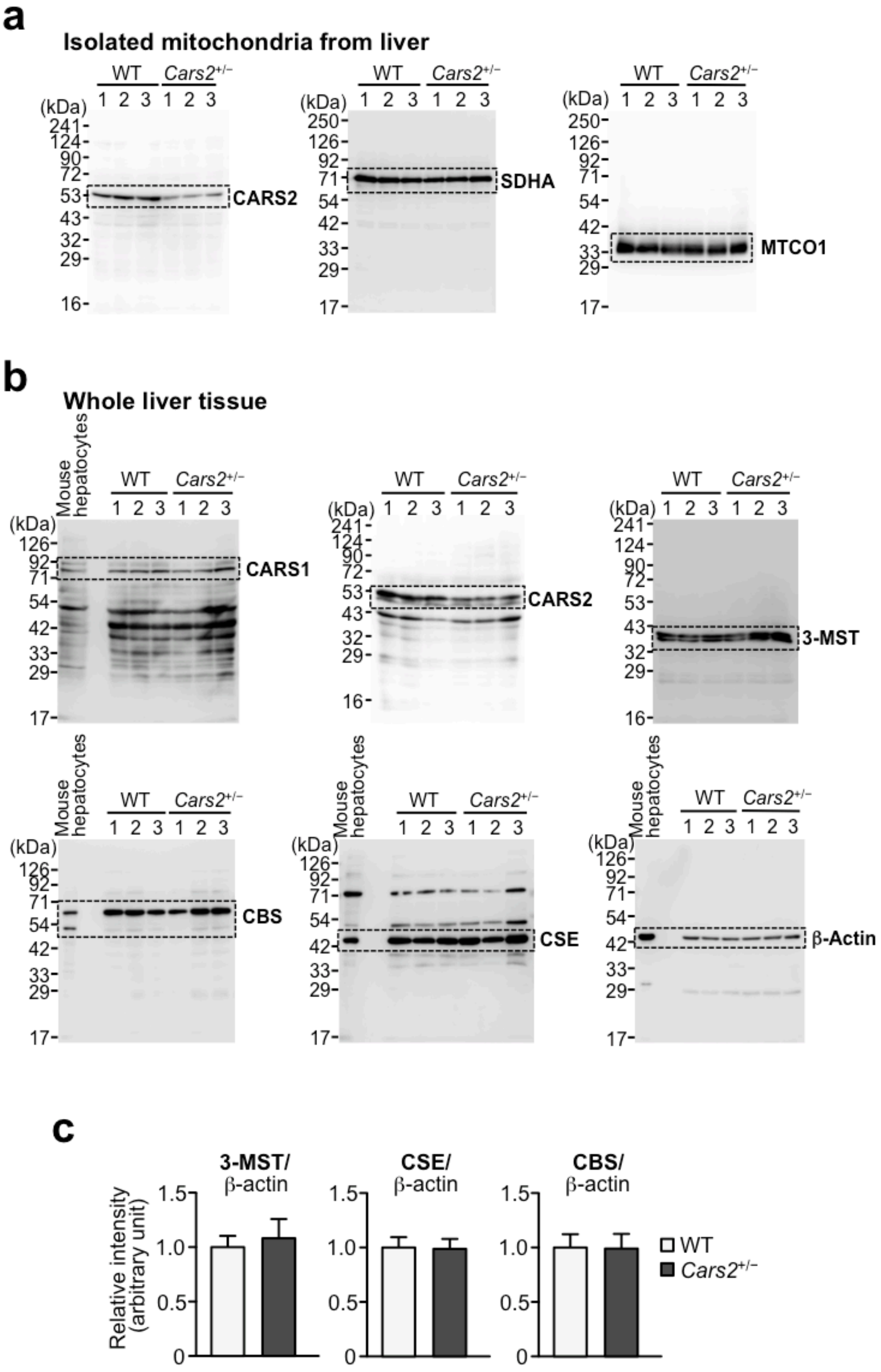

Supplementary Fig. 19. Uncropped images of the blots shown in Fig. 5. (a, b) Full uncropped images of Figs. 5c and 5e, respectively. Fig. 5 shows the areas marked by dashed lines. (**c**) Relative immunoreactive band intensity determined by densitometric analysis of the Western blots (**b**). Data are means ± s.d. (n = 3).

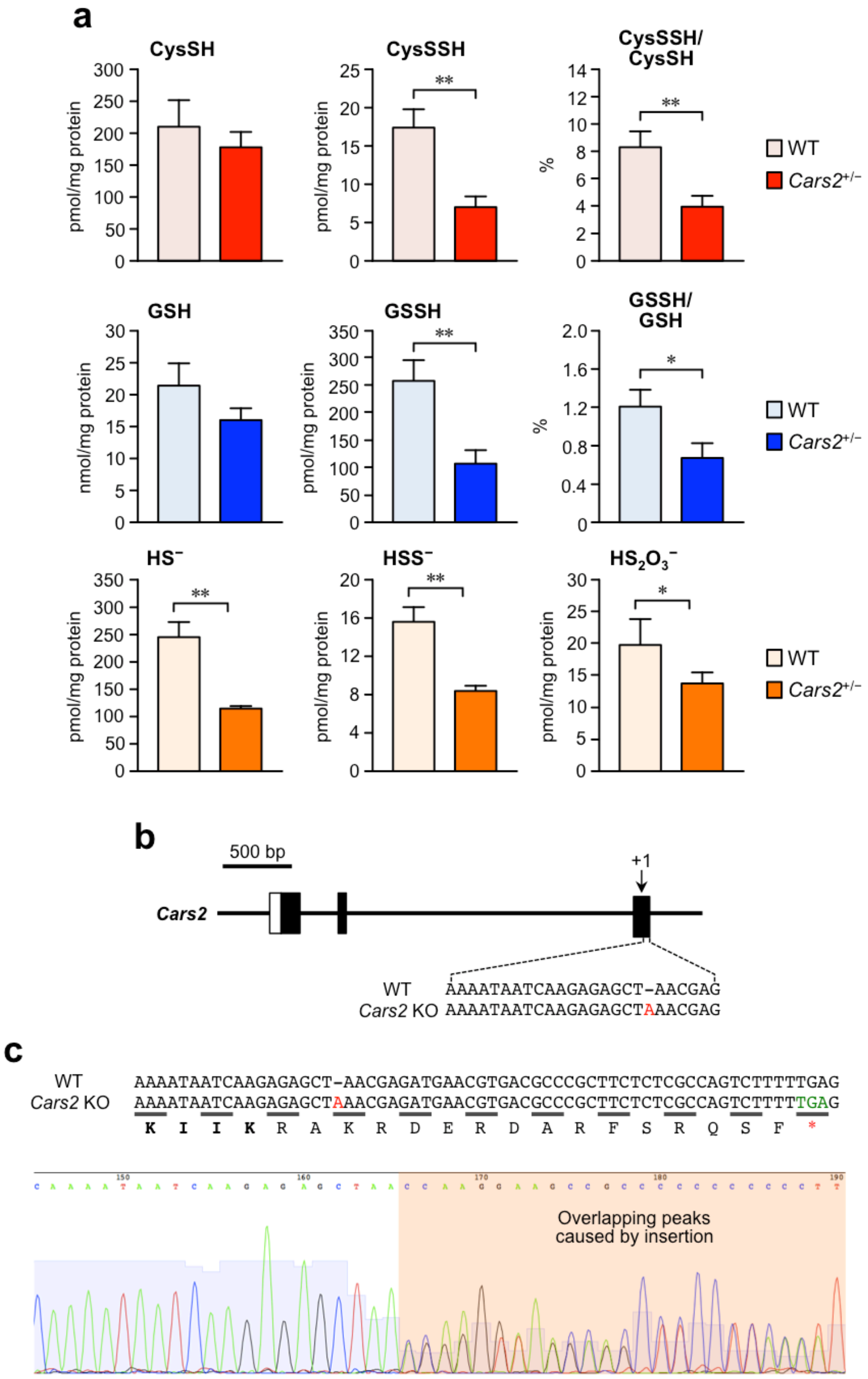

Supplementary Fig. 20. *In vivo* formation of CysSSH and other related polysulfides in WT and *Cars2*$^{+/-}$ mice (line 2). (**a**) The endogenous *in vivo* production of CysSSH and other related polysulfide compounds was identified in the liver obtained from WT and *Cars2*$^{+/-}$ littermates (line 2, 17-week-old male mice) by

means of HPE-IAM labeling LC-ESI-MS/MS analysis. Data are means ± s.d. (n = 3). *P < 0.05; **P < 0.01. (**b**) Schematic illustration of the mouse *Cars2* gene structure and sequences of the WT and mutant allele (line 2) around the target locus. A modified allele sequence obtained from the *Cars2* targeted mouse is shown below. Black letters are partial sequences of the third exon, and the 1-bp insertion is in red. **(c)** The 1-bp insertion was expected to produce a truncated CARS2 protein by introducing a stop codon (in green) just after the KIIK motif. The lower panel shows the results of direct sequencing of the *Cars2* locus around the gRNA target site. One allele received a 1-bp insertion that caused continuous overlapping peaks.

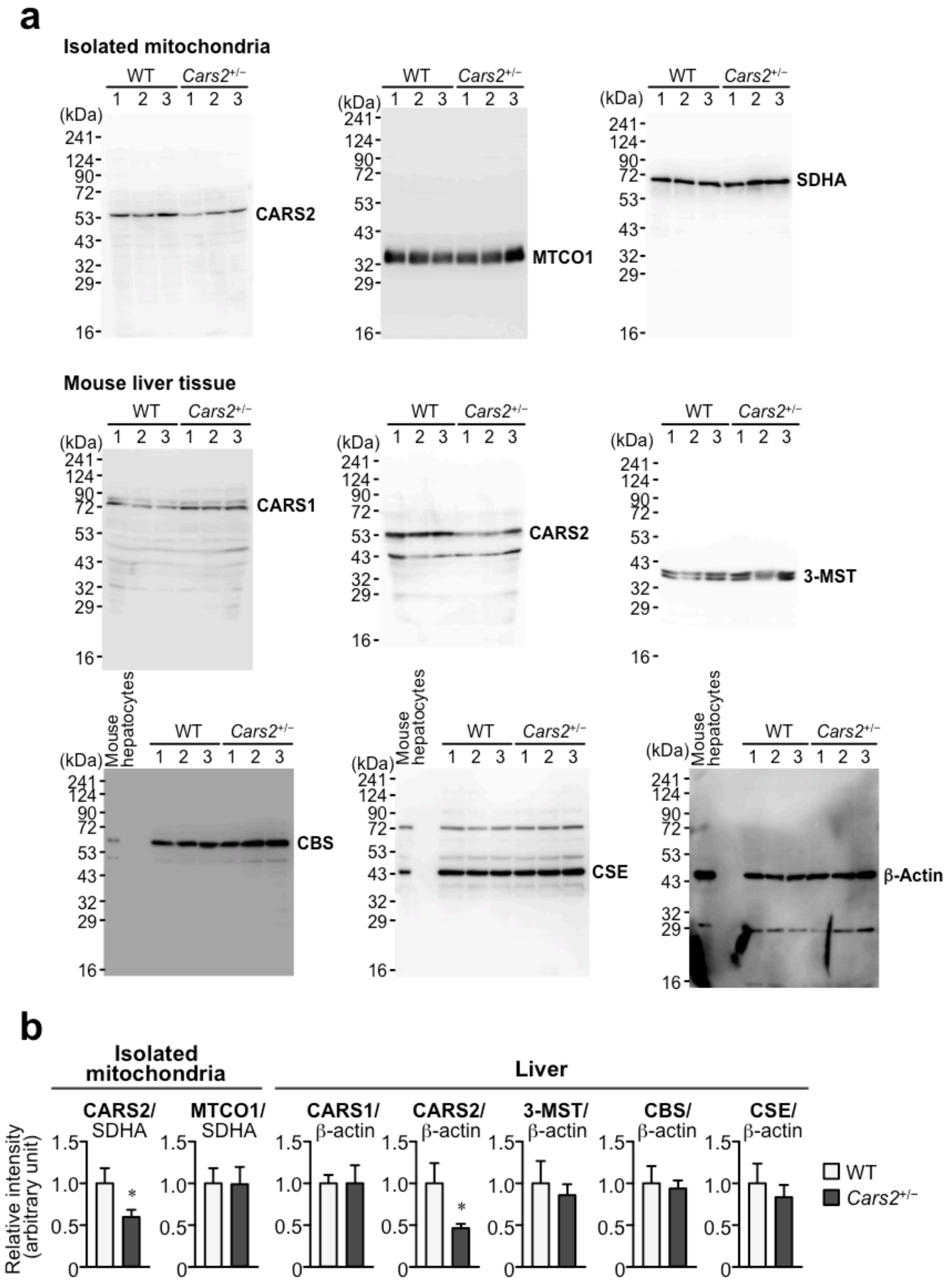

Supplementary Fig. 21. Western blotting of different persulfide-related enzymes from isolated mitochondria and mouse liver. (**a**) Mitochondria isolated from mouse liver and whole liver homogenates from WT and *Cars2*$^{+/-}$ mice (line 2) were analyzed. (**b**) Relative immunoreactive band intensity for bands in **a**. Data are means ± s.d. (n = 3). *P <0.05.

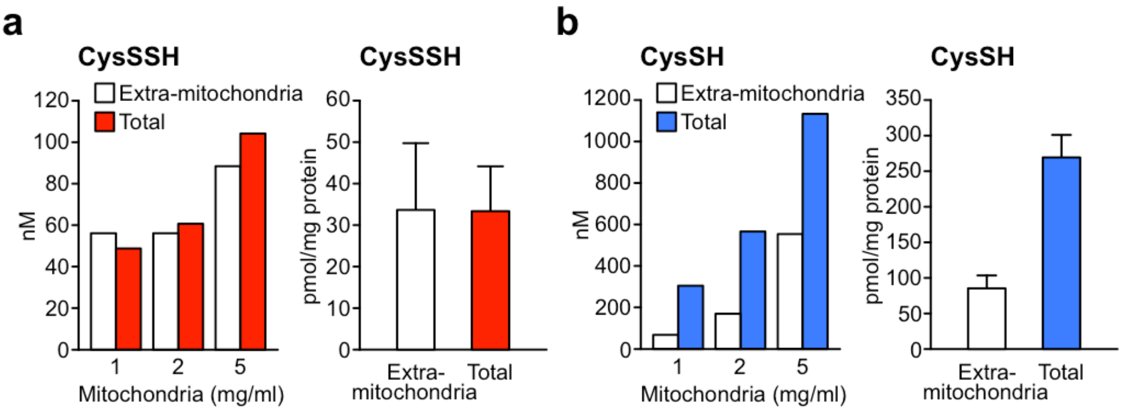

Supplementary Fig. 22. CysSSH release from mitochondria. The mitochondrial fraction isolated from mouse liver was incubated at 37 ºC for 1 h to generate CysSSH, after which CysSSH (**a**) and cysteine (CysSH) (**b**) released extramitochondrially and CysSSH and CysSH in the total mitochondrial suspension (Total) were quantified by using HPE-IAM labeling LC-ESI-MS/MS analysis. The left and right panels in **a** and **b** show the concentrations in the mitochondrial suspension and the amounts assessed per mitochondrial protein, respectively.

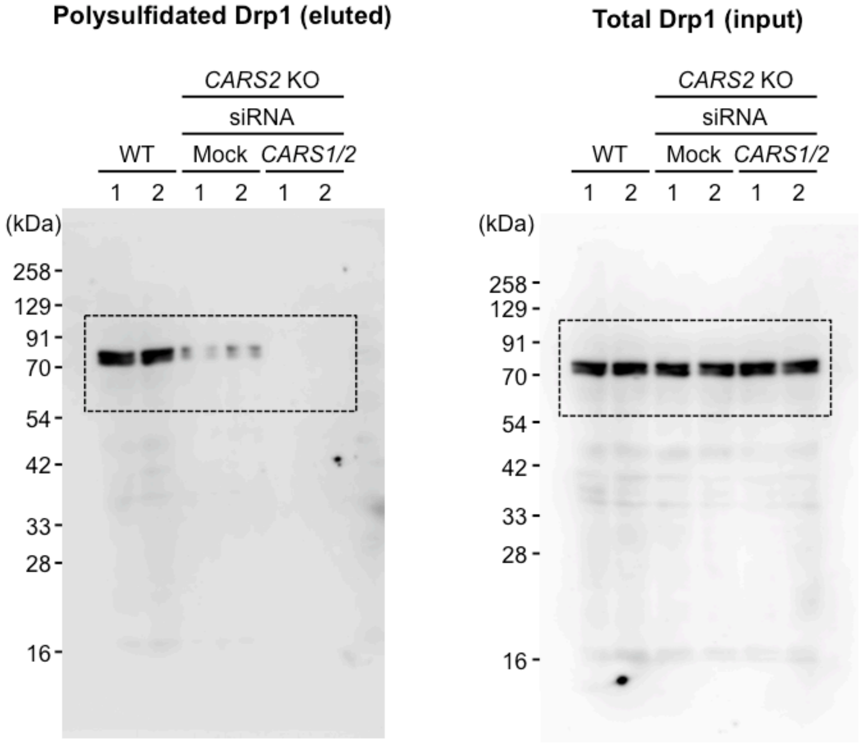

Supplementary Fig. 23. Uncropped images of the blots shown in Fig. 8d. Fig. 8d shows the areas marked by dashed lines. Reduced protein polysulfidation in *CARS2* KO with *CARS1/2* knockdown samples likely resulted in marked reduction of immunoreactivity of anti-Drp1 antibody in the biotin-PEG$_{36}$-MAL-captured samples compared with WT samples (left panel).

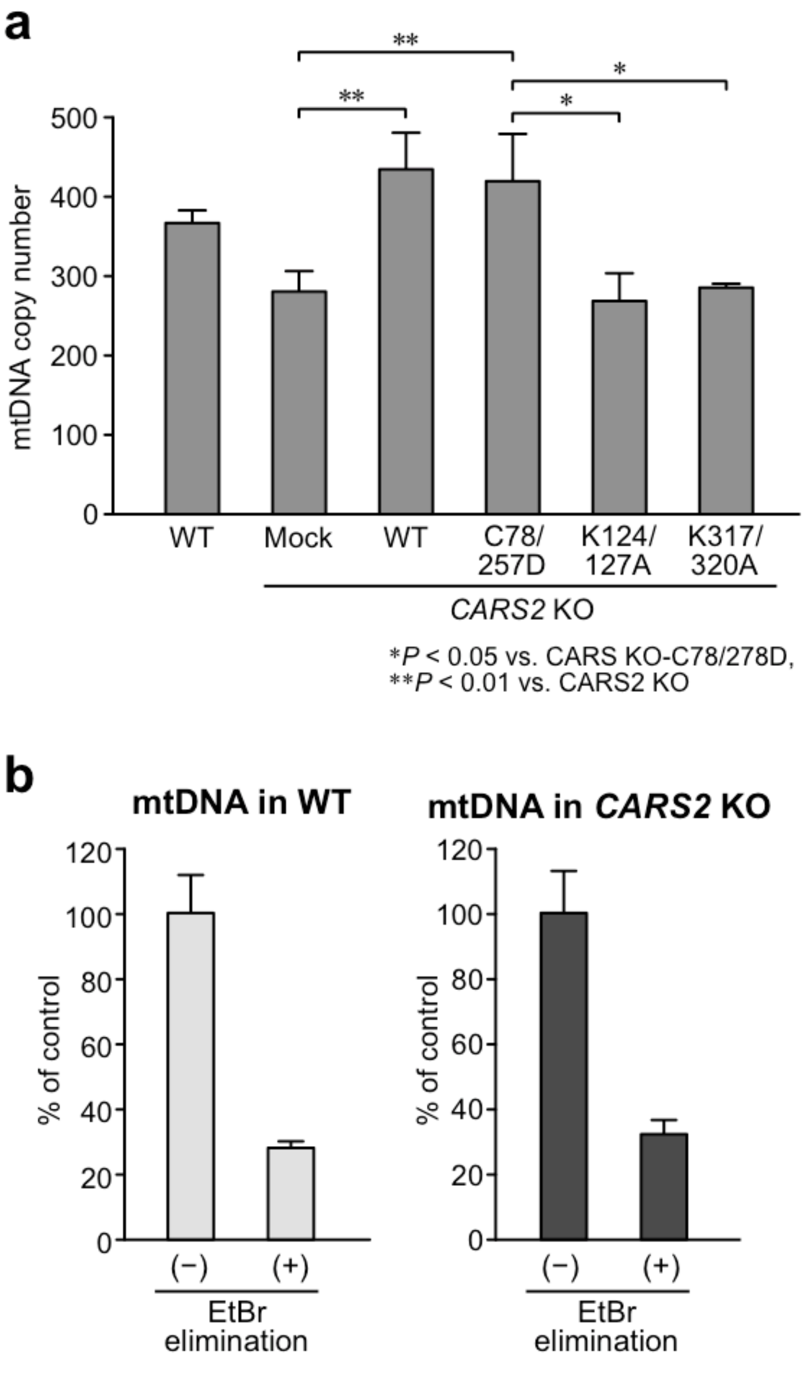

Supplementary Fig. 24. Amount of mitochondrial DNA (mtDNA) in WT and *CARS2* KO HEK293T cells. (**a**) The effect of *CARS2* KO on the amount of mtDNA in the cells was examined by measuring mtDNA amounts via quantitative real-time PCR. Data are means ± s.d. (n = 3). *P < 0.05 (vs. *CARS2* KO C78/257D); **P < 0.01 (vs. *CARS2* KO). (**b**) Changes in mtDNA in WT and *CARS2* KO HEK293T cells treated with ethidium bromide (EtBr).

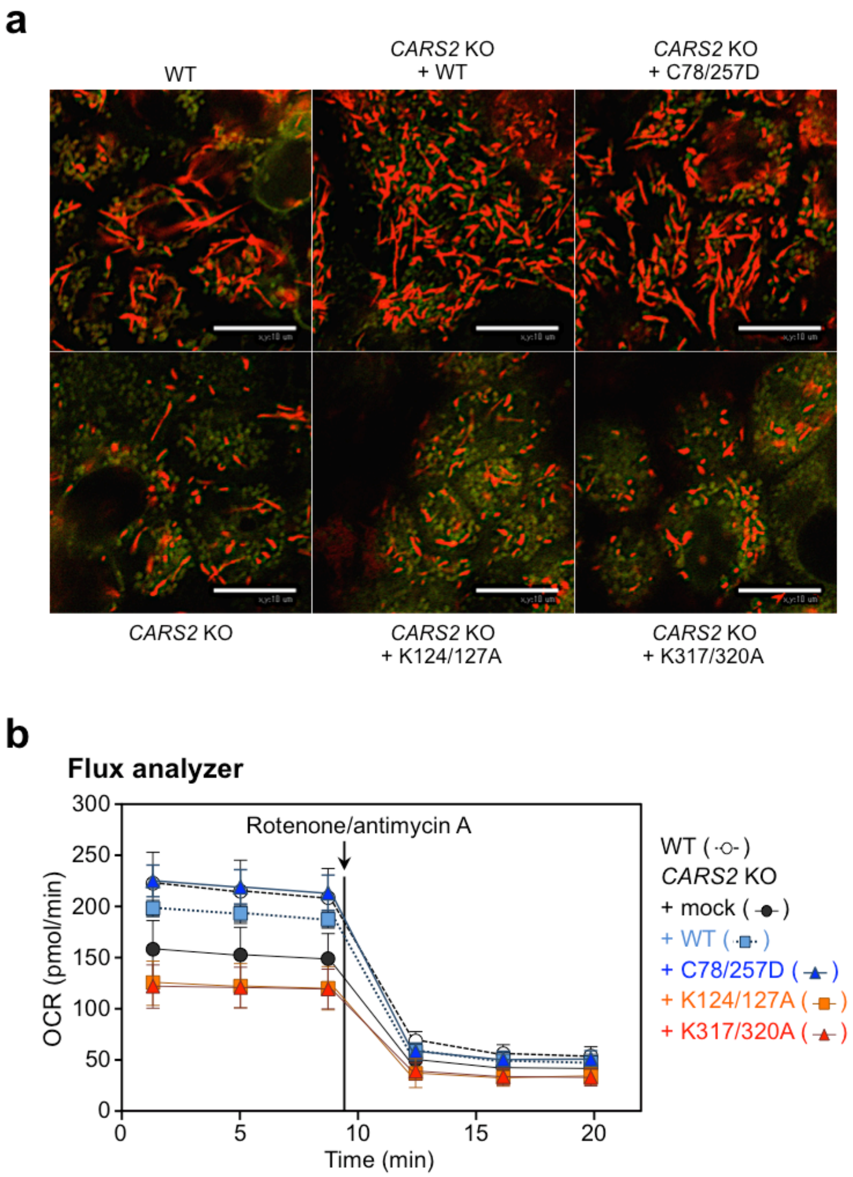

Supplementary Fig. 25. CARS2-dependent mitochondrial dynamics in bioenergetics. (**a**) Mitochondrial bioenergetics was analyzed by using JC-1 fluorescence imaging for WT and *CARS2* KO HEK293T cells with or without transfection with WT or mutant *CARS2*. Fig. 8f illustrates the results of the morphometry obtained with this imaging analysis. Scale bars, 10 μm. (**b**) Assessment of mitochondrial electron flow in HEK293T *CARS2* KO cells with or without adding back WT and C78/257D, K124/127A, and K317/320A mutants, as analyzed by measuring the oxygen consumption rate (OCR) with an extracellular flux analyzer. Time dependence of oxygen consumption before and after inhibition of mitochondrial respiration at complexes I and III by rotenone and antimycin A. Fig. 8g shows the result of the quantitative analysis of this OCR response. Data are means ± s.d. ($n = 3$).

Supplementary Table 1. Primer sets for construction of *E. coli* expression vectors

Gene	Oligonucleotides*
hADH5	GCA TCT CGA GAT GGC GAA CGA GGT TAT CAA GTG C
	GCA TCT CGA GTT AAA TCT TTA CAA CAG TTC G
hGAPDH	CAT ATG GGG AAG GTG AAG GTC GGA GT
	GGA TCC TTA CTC CTT GGA GGC CAT GT
mCARS1	AGC CAT ATG ATG GCA GGT TCC TCC GCG GA
	GAG CAT ATG TTA CTG GAG GCT GCC ATT CTG T
hCARS2	ATG CTC GAG ATG TTG AGG ACT ACG CGC GG
	ATC CTC GAG TCA GCC CGC TGA TTT TTG GT
hETHE1	GGG GAT GCA GCT GAG CCA GCG CGG CGG GTC T
	GGG GAT CCG GCA GTG GGT GTC TGC ACC CCA CA
hALDH1A1	GGC ATA TGA TGT CAT CCT CAG GC
	GGC TCG AGT GAG TTC TTC TGA GA
CysK (*Salmonella*)	GGG ATC CAG TAA GAT TTA TGA AGA TAA CTC
	GGG TCG ACT CAC TGT TGC AGT TCT TTC TCA GTA

*Restriction enzyme sites for cloning are underlined.

Supplementary Table 2. MRM parameters of various per/polysufide derivatives used for LC-ESI-MS/MS analyses

Analyte	Polarity	Precursor ion (*m/z*)	Product ion (*m/z*)	Collision energy (V)
CysS-HPE-AM	+	298.9	121.0	-29
CysS-HPE-AM*	+	299.9	121.0	-29
CysSS-HPE-AM	+	330.8	121.0	-29
CysS^{34}S-HPE-AM	+	332.8	121.0	-29
Cys^{34}S^{34}S-HPE-AM	+	334.8	123.0	-29
CysSSS-HPE-AM	+	362.8	121.0	-29
CysS^{34}S^{34}S-HPE-AM	+	366.8	121.0	-29
Cys^{34}S^{34}S^{34}S-HPE-AM	+	368.8	123.0	-29
Homocysteine-HPE-AM	+	312.9	121.0	-32
Homocysteine-HPE-AM*	+	316.9	121.0	-32
Bis-S-HPE-AM	+	388.9	121.0	-30
Bis-^{34}S-HPE-AM	+	390.9	121.0	-30
Bis-SS-HPE-AM	+	420.9	121.0	-23
Bis-^{34}S^{34}S-HPE-AM	+	422.9	121.0	-23
HS_2O_3-HPE-AM	-	290.0	208.2	14
$H^{34}S_2O_3$-HPE-AM	-	294.0	210.2	14
GS-HPE-AM	+	484.9	356.3	-18
GS-HPE-AM*	+	488.1	359.3	-18
GSS-HPE-AM	+	516.9	388.3	-18
GS^{34}S-HPE-AM	+	518.9	390.1	-18
CysS-NEM	+	246.7	158.1	-22
CysS-NEM*	+	247.7	158.1	-22
CysSS-NEM	+	278.8	190.2	-16
CysS^{34}S-NEM	+	280.8	192.2	-16
GS-NEM	+	433.8	305.0	-16
GS-NEM*	+	436.8	307.2	-16
GSS-NEM	+	465.2	336.1	-17
GS^{34}S-NEM	+	467.2	336.1	-17
GSSG	+	613.0	355.0	-23
GSSG*	+	619.0	361.0	-23
GSSSG	+	645.0	387.0	-26
GS^{34}SSG	+	647.0	389.0	-26
GSSSSG	+	677.0	339.0	-25
GS^{34}S^{34}SSG	+	681.0	341.0	-25
GSSSSSG	+	709.0	371.0	-49
GS^{34}S^{34}S^{34}SSG	+	715.0	375.0	-49

*Stable isotope-labeled derivatives.

Supplementary Table 3. Enzyme kinetics for EcCARS and CSE (rat and human)

Enzyme		K_m (μM)	V_{max} (μmol mg^{-1} h^{-1})	k_{cat}/K_m (× 10^3, M^{-1} s^{-1})
EcCARS*	WT	7.3 ± 0.9	1.4 ± 0.1	1.4 ± 0.3
	C28S	4.4 ± 0.5	1.5 ± 0.1	2.7 ± 0.3
CSE*	Rat	243.0 ± 30.0	51.1 ± 2.3	2.8 ± 0.1
	Human	354.6 ± 77.6	49.3 ± 4.0	1.9 ± 0.1

*Substrates: L-cysteine for EcCARS; L-cystine for CSE.

Supplementary Table 4. Primer sets for EcCARS and hCARS2 mutant expression vectors

Expression vector	Oligonucleotides*
pCA24N-EcCARS K73A	GAT ATC GAC GAC GCA ATC ATC AAA CGC
	GCG TTT GAT GAT TGC GTC GTC GAT ATC
pCA24N-EcCARS K76A	GAC AAA ATC ATC GCA CGC GCC AAT GAA
	TTC ATT GGC GCG TGC GAT GAT TTT GTC
pCA24N-EcCARS K266A	GTT GAC CGC GAG GCG ATG TCC AAA TCG
	CGA TTT GGA CAT CGC CTC GCG GTC AAC
pCA24N-EcCARS K269A	GAG AAG ATG TCC GCA TCG CTG GGT AAC
	GTT ACC CAG CGA TGC GGA CAT CTT CTC
pCA24N-EcCARS C28S	GGC ATG TAC GTG TCT GGA ATC ACC GTT
	AAC GGT GAT TCC AGA CAC GTA CAT GCC
pCA24N-EcCARS C209S	TGG CAC ATT GAA TCT TCG GCA ATG AAC
	GTT CAT TGC CGA AGA TTC AAT GTG CCA
pCA24N-EcCARS C28D	GGC ATG TAC GTG GAT GGA ATC ACC GTT
	AAC GGT GAT TCC ATC CAC GTA CAT GCC
pCA24N-EcCARS C209D	TGG CAC ATT GAA GAT TCG GCA ATG AAC
	GTT CAT TGC CGA ATC TTC AAT GTG CCA
pPyCAGIP-FLAG-hCARS2 C78D	TCC TGG TAT AGC GAT GGA CCA ACT GTA
	TAC AGT TGG TCC ATC GCT ATA CCA GGA
pPyCAGIP-FLAG-hCARS2 C257D	TGG CAC ATC GAG GAT TCT GCC ATC GCT
	AGC GAT GGC AGA ATC CTC GAT GTG CCA
pPyCAGIP-FLAG-hCARS2 K124/127A	ACA GAT GTA GAT GAT GCA ATC ATC GCA AGA GCC AAT GAG ATG
	CAT CTC ATT GGC TCT TGC GAT GAT TGC ATC ATC TAC ATC TGT
pPyCAGIP-FLAG-hCARS2 K317/320A	GGC AAA GAA GAA GCA ATG TCC GCA TCA TTA AAG AAC
	GTT CTT TAA TGA TGC GGA CAT TGC TTC TTC TTT GCC

*Mutant bases are underlined.

Supplementary Methods

Materials Cysteine (CysSH), glutathione (GSH), sodium hydrosulfide (NaHS), pyridoxal phosphate (PLP), ATP, tRNA (*Escherichia coli*), Lipofectamine 2000, Lipofectamine RNAiMAX, and other reagents were obtained from Nacalai Tesque (Kyoto, Japan), Wako Pure Chemical Industries (Osaka, Japan), Invitrogen (Carlsbad, CA), and Sigma-Aldrich (St. Louis, MO), and all remaining materials were from Sigma-Aldrich, unless specified otherwise. Authentic 8-nitroguanosine 3',5'-cyclic monophosphate (8-nitro-cGMP), cysteine per/polysulfides, and glutathione per/polysulfides were prepared according to the methods previously reported[1-3]. Na_2S_2 and biotin-polyethylene glycol (PEG)-conjugated 36-mer] maleimide (MAL) (biotin-PEG_{36}-MAL) were synthesized and provided by Dojindo Laboratories (Kumamoto, Japan), and pPyCAGIP-FLAG was a kind gift from Dr. Mitsuyoshi Nakao (Kumamoto University)[4]. Recombinant cystathionine γ-lyases (CSEs) (rat and human) were produced as described earlier[1]. L-[^{34}S]Cysteine was synthesized from *O*-acetyl-L-serine and ^{34}S-labeled disodium sulfide via a unique catalytic reaction of cysteine synthase (CysK), whose recombinant protein was produced as described below. Briefly, *O*-acetyl-L-serine (20 mM) was reacted with 20 mM $Na_2{}^{34}S$ in 100 mM sodium phosphate buffer (pH 7.6) in the presence of 0.05 mg/ml CysK at 37 °C for 1 h. L-[^{34}S]Cysteine was purified from the reaction mixture by means of high-performance liquid chromatography (HPLC). The specific rat polyclonal antibody for mouse CARS2 and rabbit polyslonal antibody for CSE were produced by immunizing rats and rabbits with recombinant mouse CARS2 and rat CSE proteins, respectively, and by immunoaffinity purification, as described earlier[3].

Preparation of polysulfide adducts of β-(4-hydroxyphenyl)ethyl iodoacetamide and *N*-ethylmaleimide CysSH/CysS-$(S)_n$-H and GSH/GS-$(S)_n$-H adducts with β-(4-hydroxyphenyl)ethyl iodoacetamide (HPE-IAM) (Molecular BioSciences, Boulder, CO) and *N*-ethylmaleimide (NEM) were synthesized according to our previous report[1]. In brief, 0.5 mM cysteine and GSH were reacted with 0.5 mM NaHS in the presence of 0.5 mM 1-hydroxy-2-oxo-3-(*N*-methyl-3-aminopropyl)-3-methyl-1-triazene (Dojindo Laboratories) in 10 mM Tris-HCl buffer (pH 7.4) at room temperature for 30 min, after which HPE-IAM or NEM (all at 5 mM) was added to the reaction mixture, and then the mixtures were incubated at room temperature for 20 min to form respective CysSH/CysS-$(S)_n$-H and GSH/GS-$(S)_n$-H adducts. Similarly, HPE-IAM and NEM adducts of stable isotope-labeled CysS-$(^{34}S)_n$-H or GS-$(^{34}S)_n$-H were synthesized by reacting each per/polysulfide with $NaH^{34}S$ instead of $NaH^{32}S$. Various per/polysulfide adducts thus synthesized were easily purified via the HPLC system (Prominence; Shimadzu Corporation, Kyoto, Japan), because of increased hydrophobicity conferred by the polysulfide residues, on a reverse-phase column (YMC-Triart C18 column, 50 × 2.0 mm inner diameter; YMC, Kyoto, Japan) under the following elution conditions: mobile phases A (0.1% formic acid) and B (0.1% formic acid in methanol) with a linear

gradient from 5% to 90% B for 15 min at a flow rate of 0.2 ml/min at 40 °C.

Human, mouse, and *E. coli* cDNA Full-length cDNAs of human alcohol dehydrogenase 5 (hADH5, class III; also known as *S*-nitrosoglutathione reductase), human glyceraldehyde-3-phosphate dehydrogenase (hGAPDH), human aldehyde dehydrogenase A family, member A1 (hALDH1A1), and human mitochondrial cysteinyl-tRNA synthetase (hCARS2) (pCMV6-Entry-hADH5, pCMV6-Entry-hGAPDH, pCMV6-Entry-hALDH1A1, pCMV6-Entry-hCARS2) were purchased from OriGene Technologies (Rockville, MD). Mouse CARS1 cDNA was amplified by means of polymerase chain reaction (PCR) and inserted into a pcDNA3 mammalian expression vector. Briefly, total RNA was isolated from mouse C2C12 cell lines by using the RNeasy kit (Qiagen, Hilden, Germany), and single-stranded cDNA was synthesized via reverse transcription with the PrimeScript 1st strand cDNA Synthesis Kit (Takara Bio, Shiga, Japan). The cDNA obtained was amplified by using KOD FX Neo DNA polymerase (Toyobo, Osaka, Japan) and the specific primer set (Supplementary Table 1) containing HindIII and KpnI sites for mouse CARS1 (accession, NM_001252593). The PCR protocol was as follows: cycling (25 cycles), 98 ºC for 30 s, 60 ºC for 30 s, and 68 ºC for 2 min. To generate pcDNA3-EGFP-mCARS1, the PCR products, after cleavage with HindIII and KpnI, were ligated into a pcDNA3 mammalian expression vector (Invitrogen). Open reading frames of CARSs from *E. coli*, cysteinyl-tRNA synthetase (EcCARS), with His tags at the N-termini (pCA24N-EcCARS), were obtained from the National BioResource Project at the National Institute of Genetics, Japan (NBRP-E.coli at NIG).

Construction of *E. coli* expression vectors PCR amplification of hADH5 cDNA was carried out with pCMV6-Entry-hADH5 as a template by using a primer set (Supplementary Table 1, restriction sites are underlined), and the resultant amplicon was digested with XhoI and cloned into the XhoI site of pET-15b to generate pET-15b-hADH5. To prepare a GAPDH expression vector, pCMV6-Entry-hGAPDH was used as a template for PCR amplification of hGAPDH cDNA with a primer set (Supplementary Table 1), and the amplicon was digested with NdeI and BamHI and cloned into the NdeI and BamHI sites of pET-30a(+) to generate pET-30a(+)-hGAPDH. To generate the vector pQE-70-hETHE1 expressing human ETHE1 (ethylmalonic encephalopathy 1; also known as GSSH dioxygenase), a cDNA library from A549 human lung cells corresponding to the *ETHE1* gene (mitochondrial import signal truncated) was amplified by using a primer set (Supplementary Table 1). A resultant DNA fragment was cloned downstream of the T5 promoter between the SphI and BamHI sites of the pQE70 plasmid (Qiagen). pCMV6-Entry-hALDH1A1 was used as a template for PCR amplification of hALDH1A1 cDNA with a primer set (Supplementary Table 1). The resultant amplicon was digested with NdeI and XhoI and cloned into the NdeI and XhoI sites of pET-30a(+) to generate pET-30a(+)-hALDH1A1. To produce pCA24N-EcCARS K73A, pCA24N-EcCARS K76A, pCA24N-EcCARS K266A, pCA24N-EcCARS K269A, pCA24N-EcCARS

C28S, pCA24N-EcCARS C209S, pCA24N-EcCARS C28D, pCA24N-EcCARS C209D, pCA24N-EcCARS K73/76A, pCA24N-EcCARS K266/269A, pCA24N-EcCARS C28/209S, and pCA24N-EcCARS C28/209D, site-directed mutagenesis was performed via inverse PCR with high-fidelity DNA polymerase KOD FX (Toyobo) and primer sets. pCA24N-EcCARS was used as a template for PCR amplification, and Supplementary Table 4 provides primer sets for introducing mutagenesis (mutant bases are underlined). The PCR protocol was as follows: denaturing, 94 ºC for 2 min; cycling (16 cycles), 98 ºC for 10 s, 55 ºC for 30 s, and 68 ºC for 1 min/kb. The PCR products were digested with SfiI (New England BioLabs, Ipswich, MA) and cloned into the SfiI site of pCA24N. To generate pET-15b-mCARS1, mCARS1 cDNA was PCR-amplified with pcDNA3-EGFP-mCARS1 as a template and a primer set (Supplementary Table 1). The resultant amplicon was digested with NdeI and cloned into the NdeI site of pET-15b to generate pET-15b-mCARS1. pCMV6-Entry-hCARS2 was used as a template for PCR amplification of human CARS2 cDNA, with a primer set (Supplementary Table 1), to obtain the amplicon, which was then digested with XhoI and cloned into the XhoI site of pET-15b to finally construct the pET-15b-hCARS2. To produce a CysK expression vector, *Salmonella* Typhimurium LT2 genomic DNA corresponding to CysK was obtained via PCR by using a primer set (Supplementary Table 1), whose amplicon was inserted into the BamHI and SalI sites of pQE80L (Qiagen) to produce pQE80L-cysK. All plasmids generated by using PCR were verified via DNA sequencing.

Preparation and purification of recombinant proteins Recombinant hADH5 was purified according to a previously described method with modifications[5,6]. Briefly, *E. coli* BL21 (DE3) transformed with pET-15b-hADH5 was grown at 30 ºC to an OD_{600} of 0.6-0.7 and induced by adding 0.1 mM isopropyl β-D-thiogalactopyranoside (IPTG) (Sigma-Aldrich) for 18 h at 15 ºC. The other *E. coli* cultures (hETHE1, hGAPDH, hALDH1A1) were induced by adding 1 mM IPTG and culturing them for 3 h at 37 °C. The *E. coli* cells, transformed with various expression vectors, were harvested by centrifugation; resuspended in 20 mM Tris-HCl buffer (pH 8.0) containing 10 mM 2-mercaptoethanol (2-ME), 0.25 M NaCl, 1 mg/ml lysozyme, and protein inhibitor cocktail (Nacalai Tesque); and lysed by sonication. The resultant cell lysates were centrifuged at 10,000 × *g* for 20 min, and the supernatants were loaded onto a column packed with nickel nitrilotriacetic acid (Ni-NTA) agarose (Qiagen) equilibrated in 20 mM Tris-HCl buffer (pH 8.0) containing 10 mM 2-ME and 0.25 M NaCl. The hADH5 protein was eluted with 200 mM imidazole and then dialyzed with 20 mM Tris-HCl (pH 8.0) containing 10 mM 2-ME, 10% glycerol, and 10 μM $ZnSO_4$ at 4 ºC for 18 h; the protein was stored at -80 ºC. Other proteins were purified in the same buffer but without 2-ME, glycerol, and $ZnSO_4$. After desalting by means of the NAP-5 column with 20 mM Tris-HCl buffer (pH 8.0) and 1 mM Tris(2-carboxyethyl)phosphine (TCEP) (Nacalai Tesque), proteins were stored at -80ºC. To prepare a recombinant CysK, BL21 (DE3) pLysS was transformed with pQE80L-cysK and cultured for induction by 0.5 mM IPTG for 1 h at 30 °C, after which

the CysK produced was purified by using Ni-NTA agarose and stored at -30 °C. Protein concentration was determined by using the Protein Assay CBB Solution (Nacalai Tesque), and protein purity was confirmed via SDS-PAGE.

Construction of mammalian expression vectors To generate pPyCAGIP-FLAG-hADH5, the XhoI fragment of pET-15b-hADH5 was cloned into the XhoI site of pPyCAGIP-FLAG[4]. To generate pPyCAGIP-FLAG-hCARS2, the XhoI fragment of pET-15b-hCARS2 was cloned into the XhoI site of pPyCAGIP-FLAG. The same vectors containing various mutant *hCARS2* genes were obtained via site-directed mutagenesis by using inverse PCR with high-fidelity DNA polymerase KOD FX (Toyobo) with pPyCAGIP-FLAG-hCARS2 as a template and primer sets for generation of pPyCAGIP-FLAG-hCARS2 C78/257D, K124/127A, and K317/320A. Supplementary Table 4 provides the primer sets for introducing mutagenesis (mutant bases are underlined).

Cell culture Human embryonic kidney (HEK) 293T cells (CRL-11268, American Type Culture Collection) and mouse embryonic fibroblasts (MEFs) were cultured in high-glucose Dulbecco's modified Eagle's medium (DMEM) (Sigma-Aldrich) that was supplemented with 10% fetal bovine serum (FBS) and 1% penicillin-streptomycin, under standard cell culture conditions (37 °C, humidified, 5% CO_2/95% air).

Establishment of *Adh5*$^{-/-}$ MEFs expressing FLAG-hADH5 *Adh5*$^{-/-}$ mice on a C57BL6/J genetic background were generated and maintained as described previously[7]. All mice were kept under specific-pathogen-free conditions and were treated according to the regulations of the Standards for Human Care and Use of Laboratory Animals of Tohoku University and the Guidelines for Proper Conduct of Animal Experiments of the Ministry of Education, Culture, Sports, Science, and Technology (MEXT), Japan. The Tohoku University Committee for Laboratory Animal Research approved all animal experiments. *Adh5*$^{-/-}$ MEFs were established from embryos at E13.5 and immortalized by lentiviral introduction of SV40 large T antigen. pPyCAGIP-FLAG-hADH5 was transfected into *Adh5*$^{-/-}$ MEFs via the MP-100 Microporator (Digital Bio Technology, Seoul, South Korea). Stable transformants were selected with 2 μg/ml puromycin (Invitrogen).

Analysis of protein polysulfidation via gel shift assay Various polysulfidated proteins were detected by using the biotin-PEG-MAL labeling gel shift assay (PMSA), which we recently developed[8,9] and herein describe as a modified version (Supplementary Figs. 3a and 4). For example, purified recombinant proteins were applied to the PD SpinTrap G-25 column (GE Healthcare, Little Chalfont, England) equilibrated with RIPA buffer (10 mM Tris-HCl, 1% NP-40, 0.1% sodium deoxycholate, 0.1% SDS, 150 mM NaCl, pH 7.4) to remove reductants. After the proteins were quantified, they (0.3 mg/ml) were incubated in RIPA buffer that included 1 mM biotin-PEG_{36}-MAL at 37 °C for 1 h. The mixture was subsequently incubated

with various electrophiles (3 mM each), including IAM, MBB, 5'-dithiobis(2-nitrobenzoic acid) (DTNB), 4,4'-dithiopyridine (DTP), methyl methanethiosulfonate (MMTS), 2-methylsulfonyl benzothiazole (MSBT), 2-aminosulfonyl benzothiazole (ASBT), *p*-chloromercuribenzoic acid (PCMB), NEM, and 8-nitro-cGMP, at 37 °C for 1 h, after which the proteins were heat-denatured in the presence or absence of 5% 2-ME and subjected to SDS-PAGE and CBB staining. For PMSA analysis with lysates of A549 cells, *Adh5*$^{-/-}$ MEFs expressing ADH5 and HEK293T cells were washed with ice-cold phosphate-buffered saline (PBS, pH 7.4) and collected with ice-cold RIPA buffer plus 1 mM TCEP and proteinase inhibitor cocktail (Nacalai Tesque). Cells were homogenized by 20 passages through a 26-gauge needle with a 1-ml syringe on ice and then centrifuged at 20,000 × *g* at 4 °C for 25 min. The supernatant was collected and incubated at 37 °C for 1 h in the presence of 1 mM TCEP, after which the protein was stored at -80 °C until use. After TCEP was removed with the PD SpinTrap G-25 column, cell lysate proteins (3 mg/ml) were analyzed according to the same procedure as that used for the PMSA assay for recombinant proteins, and individual proteins were detected by using Western blotting with specific antibodies.

Identification of polysulfidated proteins via the biotin-PEG-MAL capture method PMSA can be applied to a biotin-PEG-MAL capture method (Supplementary Fig. 3b) for quantitative identification of endogenous polysulfidated proteins, which are isolated by reductive treatment from the biotin-PEG-MAL-bound avidin beads, followed by specific detection with Western blotting. Briefly, cells were lysed with ice-cold RIPA buffer containing 1 mM biotin-PEG$_{36}$-MAL and a proteinase inhibitor cocktail. After whole biotinylated proteins in the lysates were captured and enriched with Streptavidin Mag Sepharose (GE Healthcare), polysulfidated proteins were collected and subjected to Western blotting for Drp1. The biotin-PEG-MAL capture method is conceptually similar to the ProPerDP method recently reported[10].

Preparation of mitochondria A mitochondrial fraction was isolated from WT and *Cars2*$^{+/-}$ mice as described previously[11]. Briefly, liver tissues were homogenized in isotonic buffer [10 mM HEPES, pH 7.4, 75 mM sucrose, 225 mM mannitol, 2 mM ethylenediaminetetraacetic acid (EDTA)] with a Teflon homogenizer for 15 strokes at 700 rpm and centrifuged at 700 × *g* for 10 min at 4 ºC. The supernatants were centrifuged again at 5,000 × *g* for 10 min at 4 ºC. The pellets were washed twice with the isotonic buffer. To examine the amount of CysSSH and cysteine released from mitochondria, the resupended mitochondria with the isotonic buffer were incubated at 37 °C for 1 h, followed by centrifugation (5,000 × *g*, 10 min, 4 ºC). The supernatants were alkylated with 1 mM HPE-IAM in RIPA buffer and were subjected to LC-ESI-MS/MS as mentioned above. To determine the amount of total CysSSH and cysteine generated by mitochondria, resupended mitochondria in isotonic buffer were incubated at 37 °C for 1 h. Mitochondria-containing mixtures were alkylated with 1 mM HPE-IAM in RIPA buffer and were subjected to LC-ESI-MS/MS as mentioned above.

Protein polysulfidation identified by LC-ESI-MS/MS and quadrupole -time-of-flight-MS CysS-$(S)_n$-H formed in various proteins were identified by means of LC-ESI-MS/MS analysis as just described and by LC-quadrupole (Q)-time-of-flight (TOF)-MS (LC-Q-TOF-MS) analysis combined with Mascot searches. Briefly, purified recombinant proteins were applied to a PD SpinTrap G-25 column equilibrated with 10 mM HEPES buffer (pH 7.5) to remove reductants, after which each protein (0.85 mg/ml) was alkylated with 6 mM HPE-IAM at 37 °C for 5 min and was digested by 1 mg/ml Pronase (Merck, Darmstadt, Germany), in 40 mM sodium acetate buffer (pH 5.5) in the presence of known amounts of isotope-labeled internal standards at 37 °C for 7 h, to produce cysteine or CysS-$(S)_n$-H. After addition of 0.1% formic acid to the Pronase digest and centrifugation, the supernatants were subjected to LC-ESI-MS/MS. To identify the sites of polysulfur formation and sulfur numbers in each protein, recombinant hADH5 or hGAPDH, with 3 mM 2-ME or 0.3 mM TCEP, was alkylated with 10 mM IAM in 20 mM Tris-HCl buffer (pH 7.5) at 37 °C for 10 min, followed by digestion with 10 μg/ml trypsin (sequencing grade, modified; Promega) at 37 °C for 30 min. The digest samples diluted with 0.1% formic acid were subjected to LC-Q-TOF-MS as previously reported[2]. LC-Q-TOF-MS was performed with an Agilent 6510 Q-TOF mass spectrometer (Agilent Technologies), with an HPLC chip-MS system consisting of a nano pump with a four-channel microvacuum degasser, and a microfluidic chip cube. A microfluidic reverse-phase HPLC-chip (Zorbax 300SB-C18; 5-μm particle size, 75 μm inner diameter, and 43 mm length; Agilent Technologies) was used to separate the tryptic digest. The nano pump generated an isocratic flow of 400 nl/min with 0.1% formic acid and 3% acetonitrile. The capillary pump was for loading samples with a mobile phase of 0.1% formic acid at 4 μl/min. The ESI-Q-TOF instrument was operated in the positive ionization mode with an ionization voltage of 1750 V and a fragmentor voltage of 175 V at 300 °C. The selected *m/z* ranges were 300-1000 Da in the MS mode, and the instrument setting was 4 s^{-1} for the MS scan rate. The exact mass numbers of the peptide fragment containing the carbamidomethyl (CAM)-cysteine residues were obtained from Mascot MS/MS ion searches of the National Center for Biotechnology Information nonredundant (NCBI nr) database via the Matrix Science Web server Mascot version 2.2. Default search parameters were the following: enzyme, trypsin; maximum missed cleavage, 1; variable modifications, CAM (C); peptide tolerance, ±1.2 Da; MS/MS tolerance, ±1.2 Da. The digested peptide fragments with the polysulfidated cysteine residues were identified on the basis of their *m/z* values calculated by adding mass numbers of sulfur atoms equivalent to polysulfide chains comprising 1−4 sulfurs.

Identification of protein polysulfidation in cells and mouse tissues by LC-ESI-MS/MS To identify protein polysulfidation produced in HEK293T cells and mouse liver, cell and tissues were homogenized with a Polytron homogenizer with RIPA buffer containing 5 mM HPE-IAM, followed by centrifugation (14,000 × *g*, 10 min, 4 °C). The addition of 5 mM HPE-IAM to the cell lysates and tissue

homogenates effectively prevented artifactual polysulfide transfer from low-molecular-weight (LMW) polysulfides to the protein thiols. The supernatants then obtained were applied to a PD SpinTrap G-25 column equilibrated with RIPA buffer to remove LMW compounds (including various LMW persulfides), after which protein (0.4 mg/ml) was digested with 3 mg/ml Pronase in the presence of 1 mM HPE-IAM for 1 h as mentioned above. After addition of 0.1% formic acid to the Pronase digest and centrifugation, the supernatants were subjected to LC-ESI-MS/MS.

Identification of PLP-binding sites in recombinant EcCARS from *E. coli* cells The PLP-binding site in EcCARS was identified according to the method previously reported with slight modifications[12]. In brief, 3.4 mg/ml EcCARS was incubated with or without 0.1, 1.0, or 10 mM PLP at 37 °C for 60 min. After reduction of EcCARS with 10 mM $NaBH_4$ for 5 min, EcCARS was alkylated with 10 mM IAM in 20 mM Tris-HCl buffer (pH 8.0) containing 6 M urea at 37 °C for 15 min. EcCARS was desalted with the PD SpinTrap G-25 column equilibrated with 20 mM Tris-HCl buffer (pH 8.0) and then digested with 10 μg/ml sequencing grade modified trypsin at 37 °C for 3 h. To detect peptide fragments containing PLP-binding Lys residues, LC-Q-TOF-MS/MS was performed. Mass lists in the form of Mascot generic files were created and used as inputs for Mascot MS/MS ion searches of the NCBI nr database via the Matrix Science Web server Mascot version 2.2. Default search parameters were the following: enzyme, trypsin; maximum missed cleavage, 1; variable modifications, Carbamidomethyl (C) and PyridoxalPhosphate H2 (K); peptide tolerance, ±1.2 Da; MS/MS tolerance, ±1.2 Da.

Quantification of PLP in recombinant EcCARS and human CARS2 The mount of PLP was identified as a PLP-DNPH (2,4-dinitrophenylhydrazine) adduct extracted from recombinant EcCARS and human CARS2 by the trapping reaction with DNPH according to the method previously reported with slight modifications[13]. In brief, wild-type and each lysine mutants of EcCARS (50 μM each) was treated with 50 μM PLP in 30 mM HEPES buffer (pH 7.5) at 37 °C for 1 h, after which reaction mixture was applied to the PD SpinTrap G-25 column equilibrated with HEPES buffer (pH 7.5) to remove free forms of PLP (protein unbound). Recovered 15 μM each EcCRAS was reacted with 2 mM DNPH to extract PLP and form PLP-DNPH adduct in 30 mM HEPES buffer (pH 7.5) at 37 °C for 1 h, followed by quantification by LC-ESI-MS/MS analysis (MRM transition, 428 > 148; collision energy, -32 V). Recombinant human CARS2 (15 μM) was treated with 10, 50, 100 μM PLP as described above. PLP-DNPH adduct standard was synthesized by 1 mM PLP and 10 mM DNPH were reacted in 30 mM HEPES buffer (pH 7.5) at 37 °C for 1 h, after which PLP-DNPH adducts was purified via the HPLC system.

Computational modeling of the three-dimensional structure of PLP-bound EcCARS Molecular docking of PLP to EcCARS (PDB ID: 1LI5) was performed with SwissDock (http://www.swissdock.ch)[14]. The docking results were visualized with

PyMOL (https://www.pymol.org).

Protein synthesis by means of the cell-free PUREfrex system An EcCARS-deficient PUREfrex system was purchased from GeneFrontier (Tokyo, Japan). The genes coding hADH5, hGAPDH, hETHE1, and hALDH1A1 for the PUREfrex system were prepared via PCR according to the manufacturer's instruction, with pET-15b-hADH5, pET-30a(+)-hGAPDH, pQE-70-hETHE1, and pET-30a(+)-hALDH1A1 as templates. The amplified DNAs were purified by using a PCR isolation system (Viogene, Taipei, Taiwan). In brief, experimental conditions for the EcCARS enzymatic reaction were optimized by using various concentrations of EcCARS added to the EcCARS-deleted reaction mixture for the *in vitro* protein synthesis according to original instructions of the manufacturer. The DNA template concentrations for ADH5, GAPDH, ETHE1, and ALDH1A1 were 6, 18, 6, and 9 ng/μl, respectively. All proteins were synthesized by using the PUREfrex system in the presence or absence of various EcCARS proteins including WT and K73A, K76A, K266A, K269A, K73/76A, K266/269A, C28S, C209S, C28/209S, C28D, C209D, and C28/209D mutants. The synthesized proteins were heat-denatured and separated via SDS-PAGE and detected by Western blotting with a cocktail of different antibodies against hADH5, hGAPDH, hETHE1, and hALDH1A1.

Western blotting To detect mitochondrial proteins, cells were washed twice with PBS and were then solubilized with lysis buffer [50 mM HEPES-KOH, 150 mM NaCl, 2% 3-[(3-cholamidopropyl)dimethylammonio]-1-propanesulfonate, 1% lithium dodecyl sulfate, 10% glycerol, 2 mM EDTA, 1 mM dithiothreitol (DTT), and protein inhibitor cocktail, pH 8.0]. Cell lysate proteins were heat-denatured and separated via SDS-PAGE followed by transfer to polyvinylidene fluoride membranes (Immobilon-P) (Merck Millipore, Watford, UK). Membranes were blocked with Blocking One (Nacalai Tesque) or TTBS containing 3% skim milk (Nacalai Tesque), after which they were incubated with antibodies in TTBS containing 5% skim milk at 4 ºC overnight. Antibodies used in Western blotting included the following: anti-DDDDK-tag (1:4000 dilution) (No. M185-3L, MBL, Nagoya, Japan), anti-GAPDH (1:5000 dilution) (No. FL-3350, Santa Cruz Biotechnology, Dallas, TX), anti-ETHE1 (1:5000 dilution) (No. sc-393869, Santa Cruz Biotechnology), anti-ALDH1A1 (1:5000 dilution) (No. sc-166362, Santa Cruz Biotechnology), anti-CARS1 (1:5000 dilution) (No. HPA002383, Sigma-Aldrich), anti-CARS2 (1:5000 dilution) (No. HPA043935, Sigma-Aldrich), anti-mouse CARS2 (1:5000 dilution) (produced here), anti-β-actin (1:5000 dilution) (No. sc-1615, Santa Cruz Biotechnology), anti-CBS (1:5000 dilution) (No. 3E1A, bnova, Taipei, Taiwan), anti CSE (1:5000 dilution) (produced here), anti-3MST (1:3000 dilution) (No. sc-376168, Santa Cruz Biotechnology) anti-MTCO1 (1:5000 dilution) (No. ab14705, Abcam, Cambridge, UK), anti-SDHA (1:5000 dilution) (No. ab14715, Abcam), anti-Drp1 (1:1000 dilution) (No. sc-32898, Santa Cruz Biotechnology), and anti-ADH5 (1:5000 dilution), prepared as we described elsewhere[3]. Membranes were washed three times in TTBS and then incubated with a horseradish

peroxidase-conjugated secondary antibody (1:5000 dilution) for 1 h at room temperature. After the membranes were washed three times in TTBS, immunoreactive bands were detected via a chemiluminescence reagent (ECL Prime Western Blotting Detection Reagent; GE Healthcare) with a luminescent image analyzer (ImageQuant LAS 500; GE Healthcare). Densitometric analyses were performed to quantify these bands, with the signal intensity of the Western blotting images measured via ImageJ software. See Supplementary Figs. 16, 19, and 23 for uncropped blots.

Immunocytochemistry and transmission electron microscopy for assessment of mitochondrial morphology To investigate mitochondrial morphology under several experimental conditions with WT and *CARS2* knockout (KO) cells, we performed immunocytochemistry with anti-TOMM20 (translocase of outer mitochondrial membrane 20) antibodies (No. Ab56783, Abcam) and anti-CARS2 antibody. Briefly, cultured WT or *CARS2* KO HEK293T cells were plated in 8-well multichamber Millicell slides (Millipore) coated with polyethylene imine (PEI), with the cells being treated or untreated with various *CARS2* vectors, and the slides were fixed with 4% paraformaldehyde solution at room temperature for 15 min. After PBS washes, cells were permeabilized with 0.5% Triton X-100 at room temperature for 10 min and washed with PBS. To block nonspecific antigenic sites, cells were incubated with 1% bovine serum albumin (BSA) (Sigma-Aldrich) at room temperature for 1 h. Cultured cells were then incubated at room temperature for 1 h with the primary antibodies (10 μg/ml) in PBS with 1% BSA, after which they were rinsed five times with PBS and incubated for 1 h at room temperature with Alexa Fluor 555 goat anti-mouse IgG (H+L) (No. A21424, Thermo Fisher Scientific, Rockland, IL) and Alexa Fluor 488 goat anti-rabbit IgG (H+L) (No. A11034, Thermo Fisher Scientific) in PBS with 1% BSA. Cultured cells were washed with PBS, covered with ProLong Gold Antifade Reagent (Thermo Fisher Scientific), and examined with a Nikon EZ-C1 confocal laser microscope. Images were digitized and stored in PICT format by using a Color Chilled 3CCD Camera C5810 (Hamamatsu Photonics K.K., Shizuoka, Japan). We used ImageJ software for image processing and quantification. 4',6'-Diamindino-2-phenylindole (Thermo Fisher Scientific) served as a specific stain for the nucleus. Mitochondrial morphology was also examined by means of transmission electron microscopy (TEM), as described previously[15]. WT cells or *CARS2* KO cells were plated in 6-well plates coated with PEI. *CARS2* KO cells were transfected with WT *flag-hCARS2* and various mutants. Cells were fixed with 2% glutaraldehyde/2% paraformaldehyde in PBS at room temperature for 15 min, followed by examination with a Hitachi H-7100S electron microscope.

Quantification of mtDNA mtDNA was quantified by using nuclear DNA (nDNA) content as a standard. Total genomic DNA including mtDNA was isolated by means of the QIAamp DNA Mini Kit (Qiagen). The relative abundance of mtDNA and nDNA in the total genomic DNA was quantified by qPCR with the CFX Connect Real-Time System (Bio-Rad Laboratories, Hercules, CA) and SsoAdvanced Universal

SYBR Green Supermix (Bio-Rad Laboratories), with the Human Mitochondrial DNA (mtDNA) Monitoring Primer Set (Takara Bio). The following PCR protocol was used: 95 ºC for 4 min followed by 40 cycles each at 95 ºC for 15 s and 60 ºC for 30 s. The value of the threshold cycle number (Ct) of the mitochondrial genes and the nuclear genes was determined for each individual quantitative PCR run. The ΔCt [Ct (mitochondrial gene) - Ct (nuclear gene)] represents the relative abundance. The quantitative results were expressed as the copy number of mtDNA/cell by $2^{\Delta Ct}$.

GTP-agarose pulldown assay The GTP-agarose pulldown assay was performed according to the protocol of Gawlowski *et al.* with slight modification[16]. Briefly, cultured HEK293T cells with or without CARS2 expression were washed with ice-cold PBS and lysed in GTP-binding buffer (50 mM HEPES, 150 mM NaCl, 50 mM NaF, 1.5 mM $MgCl_2$, 1 mM EGTA, 10% glycerol, and 1% Triton X-100, pH 7.4). The lysate was centrifuged (16,000 × *g* for 15 min at 4 ºC), and an aliquot of the supernatant (300 μg of protein) was incubated with 30 μl of GTP-agarose beads (Sigma-Aldrich) equilibrated in GTP-binding buffer for 1 h at room temperature. The beads were centrifuged (1,000 × *g* at 4 ºC for 1 min) and washed twice with GTP-binding buffer. The GTP-bound proteins were eluted with 2× Laemmli buffer with 20 mM DTT, and the supernatants were subjected to SDS-PAGE. Separated proteins were electrotransferred to polyvinylidene fluoride membranes, and membrane-bound proteins were detected by using Western blotting with anti-Drp1 antibody as mentioned above.

Supplementary References

1. Ida, T. *et al*. Reactive cysteine persulfides and S-polythiolation regulate oxidative stress and redox signaling. *Proc. Natl. Acad. Sci. USA* **111**, 7606-7611 (2014).
2. Nishida, M. *et al*. Hydrogen sulfide anion regulates redox signaling via electrophile sulfhydration. *Nat. Chem. Biol.* **8**, 714-724 (2012).
3. Sawa, T. *et al*. Protein S-guanylation by the biological signal 8-nitroguanosine 3',5'-cyclic monophosphate. *Nat. Chem. Biol.* **3**, 727-735 (2007).
4. Aoto, T., Saitoh, N., Sakamoto, Y., Watanabe, S. & Nakao, M. Polycomb group protein-associated chromatin is reproduced in post-mitotic G_1 phase and is required for S phase progression. *J. Biol. Chem.* **283**, 18905-18915 (2008).
5. Sanghani, P. C. *et al*. Kinetic mechanism of human glutathione-dependent formaldehyde dehydrogenase. *Biochemistry* **39**, 10720-10729 (2000).
6. Hurley, T. D., Edenberg, H. J. & Bosron, W. F. Expression and kinetic characterization of variants of human $\beta_1\beta_1$ alcohol dehydrogenase containing substitutions at amino acid 47. *J. Biol. Chem.* **265**, 16366-16372 (1990).
7. Deltour, L., Foglio, M. H. & Gregg, D. Metabolic deficiencies in alcohol dehydrogenase *Adh1*, *Adh3*, and *Adh4* null mutant mice. Overlapping roles of Adh1 and Adh4 in ethanol clearance and metabolism of retinol to retinoic acid. *J. Biol. Chem.* **274**, 16796-16801 (1999).
8. Jung, M. *et al*. Protein polysulfidation-dependent persulfide dioxygenase activity of ethylmalonic encephalopathy protein 1. *Biochem. Biophys. Res. Commun.* **480**,180-186 (2016).
9. Kasamatsu, S. *et al*. Redox signaling regulated by cysteine persulfide and protein polysulfidation. *Molecules* **21**, 1721 (2016); doi:10.3390/molecules21121721.
10. Dóka, É. *et al.* A novel persulfide detection method reveals protein persulfide- and polysulfide-reducing functions of thioredoxin and glutathione systems. *Sci. Adv.* **2**, e1500968 (2016).
11. Wei, F. Y. *et al.* Cdk5rap1-mediated 2-methylthio modification of mitochondrial tRNAs governs protein translation and contributes to myopathy in mice and humans. *Cell Metab*. **21**, 428-442 (2015).
12. Mashalidis, H. E. *et al.* Rv2607 from Mycobacterium tuberculosis is a pyridoxine 5'-phosphate oxidase with unusual substrate specificity. *PLoS One.* **6**, e27643 (2011)
13. Simon, E. S. & Allison, J. Determination of pyridoxal-5'-phosphate (PLP)-bonding sites in proteins: a peptide mass fingerprinting approach based on diagnostic tandem mass spectral features of PLP-modified peptides. *Rapid Commun. Mass Spectrom*. **23**, 3401–3408 (2009).
14. Grosdidier, A., Zoete, V. & Michielin, O. SwissDock, a protein-small molecule docking web service based on EADock DSS. *Nucleic Acids Res*. **39**, W270–W277 (2011).
15. Wei, F. Y. *et al.* Deficit of tRNA(Lys) modification by Cdkal1 causes the development of type 2 diabetes in mice. *J. Clin. Invest.* **121**, 3598-3608 (2011).

16. Gawlowski, T. *et al*. Modulation of dynamin-related protein 1 (DRP1) function by increased *O*-linked-β-*N*-acetylglucosamine modification (*O*-GlcNAc) in cardiac myocytes. *J. Biol. Chem.* **287**, 30024-30034 (2012).
